# Comprehensive analyses of solute carrier family members identify SLC12A2 as a novel therapy target for colorectal cancer

**DOI:** 10.1038/s41598-024-55048-y

**Published:** 2024-02-23

**Authors:** Dan-yang Chen, Yang-yang Zhang, Hai-hang Nie, Hai-zhou Wang, Pei-shan Qiu, Fan Wang, Ya-nan Peng, Fei Xu, Qiu Zhao, Meng Zhang

**Affiliations:** 1 Department of Gastroenterology, Zhongnan Hospital of Wuhan University, No. 169, Donghu Road, Wuchang District, Wuhan, 430071 Hubei Province China; 2 Hubei Clinical Center and Key Lab of Intestinal and Colorectal Diseases, Wuhan, 430071 China; 3 Department of Gastroenterology, Shandong Provincial Hospital Affiliated to Shandong First Medical University, 250021 Jinan, China

**Keywords:** Solute carrier family, Colorectal cancer, Prognostic model, SLC12A2, Cancer genomics, Cancer metabolism, Gastrointestinal cancer, Oncogenes, Cancer

## Abstract

As the largest transporter family impacting on tumor genesis and development, the prognostic value of solute carrier (SLC) members has not been elucidated in colorectal cancer (CRC). We aimed to identify a prognostic signature from the SLC members and comprehensively analyze their roles in CRC. Firstly, we downloaded transcriptome data and clinical information of CRC samples from GEO (GSE39582) and TCGA as training and testing dataset, respectively. We extracted the expression matrix of SLC genes and established a prognostic model by univariate and multivariate Cox regression. Afterwards, the low-risk and high-risk group were identified. Then, the differences of prognosis traits, transcriptome features, clinical characteristics, immune infiltration and drug sensitivity between the two groups were explored. Furthermore, molecular subtyping was also implemented by non-negative matrix factorization (NMF). Finally, we studied the expression of the screened SLC genes in CRC tumor tissues and normal tissues as well as investigated the role of SLC12A2 by loss of function and gain of function. As a result, we developed a prognostic risk model based on the screened 6-SLC genes (SLC39A8, SLC2A3, SLC39A13, SLC35B1, SLC4A3, SLC12A2). Both in the training and testing sets, CRC patients in the high-risk group had the poorer prognosis and were in the more advanced pathological stage. What’s more, the high-risk group were enriched with CRC progression signatures and immune infiltration. Two groups showed different drug sensitivity. On the other hand, two distinct subclasses (C1 and C2) were identified based on the 6 SLC genes. CRC patients in the high-risk group and C1 subtype had a worse prognosis. Furthermore, we found and validated that SLC12A2 was steadily upregulated in CRC. A loss-of-function study showed that knockdown of SLC12A2 expression restrained proliferation and stemness of CRC cells while a gain-of-function study showed the contrary results. Hence, we provided a 6-SLC gene signature for prognosis prediction of CRC patients. At the same time, we identified that SLC12A2 could promote tumor progression in CRC, which may serve as a potential therapeutic target.

## Introduction

Colorectal cancer (CRC) is one of the most prevalent cancers worldwide^1,2^. Although new treatment options, such as targeted therapy and immunotherapy, are developed, the average of five-year survival rate for patients with advanced CRC is still less than 15%^2,3^. To make matters worse, the incidence of CRC has risen dramatically in people under the age of 50, with men aged 20–49 leading the way in CRC mortality during 2012–2016^4^. Due to the heterogeneity of colorectal cancer, it’s necessary to identify more effective biomarkers for prognostic prediction and personalized treatment^5^.

The solute carrier (SLC) superfamily, mainly situated on cellular and organelle membranes, is the largest transporter family, whose core functions are to facilitate the exchange of soluble substrates across lipid bilayers^6^. Depending on electrochemical or ion gradients, SLC proteins can transport a diverse range of substrates including carbohydrates, amino acids, fatty acids, vitamins and inorganic ions^7^, thereby playing critical roles in essential physiological processes. Dysregulation of specific SLC proteins has been closely linked to numerous diseases such as cancer, diabetes mellitus and hypertension^8^.

The functions of SLC proteins involve in mostly hallmarks of cancer^9,10^. Metabolism reprogramming of cancer cell relies on all kinds of nutrients transportation, thus providing enough energy for their sustaining proliferation^6,11^. Aerobic glycolysis is a special metabolic way of cancer cells; therefore, the transporters of glucose and lactic acid are core for the survival. It has been repeatedly observed that glucose transporter GLUT1/*SLC2A1* and lactic acid transporter MCT1/*SLC16A1*, MCT2/*SLC16A7*, MCT4/*SLC16A3* are upregulated in multiple tumor types and participate in tumor genesis and development^9^. SLC7A11, commonly known as xCT, contributes to maintain cellular redox homeostasis via importing extracellular cystine for glutathione biosynthesis^12^. SLC7A11 is overexpressed in many types of cancers and its overexpression promotes tumor progression partly through suppressing ferroptosis, a kind of programmed cell death induced by iron-dependent lipid peroxides^9,13^. The above SLC members have become promising potential targets for cancer therapy. What’s more, nutrient limitation frequently happens during tumor development, and the ability of cancer cells to adapt to a series of harsh conditions is the key factor to sustain survival^11^. A study demonstrated that the ability of cancer cells to survive from glutamine depletion depended on the aspartate/glutamate transporter GLAST/*SLC1A3*^14^. In addition, some SLC transporters contribute to deliver certain drugs to cancer cells, suggesting that they can be novel targets for increasing chemotherapy sensitivity and conquering drug resistance^15,16^. Although the importance of the SLC proteins we have come to realize, the role of most SLC members in cancer has not been well clarified.

In the present study, we developed a prognostic risk model based on SLC family genes by performing univariate and multivariate Cox regression. The low-risk and high-risk group were identified in the training and testing datasets of CRC. Then, we revealed the prognosis traits, transcriptome features, clinical characteristics, immune infiltration and drug sensitivity between the two groups. Furthermore, molecular subtyping was also implemented and validated according to the selected SLC genes. Finally, we validated the function of SLC12A2 in CRC by loss of function and gain of function.

## Methods

### Data source and processing

The dataset GSE39582, comprising 585 colorectal cancer (CRC) samples, was obtained from the Gene Expression Omnibus (GEO) database (http://www.ncbi.nlm.nih.gov/geo/). Similarly, the TCGA COAD dataset consisted of 512 CRC samples extracted from the Genomic Data Commons (GDC) data portal (https://portal.gdc.cancer.gov/). Exclusion criteria were applied to remove normal samples, duplicate samples, and those lacking key clinical features. After rigorous screening, a total of 556 samples remained in GSE39582 and 435 patients were included in TCGA COAD.

### Construction of the prognosis-related SLC gene signature

SLC family genes were identified based on the Human Gene Database (https://www.genecards.org/). Initially, univariate Cox regression analysis was conducted using the "survival" R package in both GSE39582 and TCGA COAD datasets. A *p*-value threshold of < 0.05 was applied to identify prognostically relevant SLC family genes in both datasets. Subsequently, GSE39583 was designated as the training set while TCGA COAD served as the test set for further analysis. Multivariate Cox regression analysis was then performed to determine the regression coefficients associated with prognosis for SLC genes across both datasets. The risk score for each sample was calculated using the formula: Risk score = $\sum_{i=1}^{N}{Exp}_{i}*{\beta }_{i}$, where "Exp" represents mRNA expression and "β" denotes the corresponding regression coefficient. Based on median risk scores, samples from GSE39582 and TCGA COAD datasets were categorized into low-risk and high-risk groups respectively. Survival analyses using Kaplan–Meier method along with log-rank test were conducted to compare outcomes between these groups. Additionally, ROC curves were plotted using "survivalROC" R package.

### Development of a prognostic nomogram for CRC patients

Individual survival probabilities were predicted by creating nomogram containing clinical features by the " rms " R package. Calibration curves predicting 1- and 3-year survival were plotted to assess the consistency of the nomogram with actual survival times.

### Differentially expressed gene (DEG) and functional enrichment analyses

The DEGs between the high-risk and low-risk groups was calculated using the "limma" *R* package, and the significant DEGs was selected by setting the adjusted *P*-value < 0.05 and |log2FC|> 0.5. Then, Gene Ontology (GO) analysis and Kyoto Gene Encyclopaedia (KEGG) analysis were performed by the "clusterProfiler" R package. Encyclopedia of Genomes (KEGG) analysis. The results were visualised by the "ggplot2" R package.

### Estimation of immune infiltration and cancer progression

First, the ESTIMATE algorithm was utilized to calculate immune score, mesenchymal score, and tumor purity, which reflect the enrichment of gene signatures in immune and mesenchymal cells^17^. Gene set variation analysis (GSVA), a nonparametric unsupervised method for gene set enrichment based on transcriptomic data^18^, was employed to obtain scores for pathways or a signature. We obtained 17 immune cell types from previously published studies as a gene signature. Relevant labels associated with CRC progression were downloaded from the KEGG database. Subsequently, each sample was analyzed using the "GSVA"R package to derive a corresponding signature score. Differential analyses were conducted using the 'limma' R package based on feature cores. The results were visualized using the "ComplexHeatmap" R package.

### Prediction of drug sensitivity

Two commonly used chemotherapy drugs (5-Fluorouracil and Cisplatin) and one targeted drug (Cetuximab) for CRC treatment were selected for the therapeutic response prediction using the“pRRophetic” R package based on the Genomics of Drug Sensitivity in Cancer (GDSC) (https://www.cancerrxgene.org/). The half-maximal inhibitory concentration (IC50) predicted for patients in training set and testing set was used to assess differential drug sensitivity. For prediction of immunotherapy response, the immunophenoscore (IPS) for COAD patients was downloaded from the cancer immunome atlas (TCIA) database (https://tcia.at/home).

### Classification based on the prognosis-related SLC genes

Non-negative matrix factor (NMF) clustering analysis of prognostically relevant SLC genes. The unsupervised NMF clustering method was implemented on the training and test sets by the "NMF" R package^19^. The "brunet" option was selected as the standard, and total of 30 iterations were performed. The optimal number of clusters was determined based on co-occurrence, dispersion, and contour index analyses. Expression differences between subtypes were assessed using principal component analysis (PCA).

### Clinical samples

Clinical colorectal cancer specimens and their corresponding non-tumor tissues were collected from Zhongnan Hospital of Wuhan University and diagnosed by the Department of Pathology. Written informed consent was obtained from the patients. This project was approved by the Ethics Committee of Zhongnan Hospital of Wuhan University (protocol #2023015 K). We confirmed that all methods were carried out in accordance with the guidelines and regulations of the Ethics Committee of Zhongnan Hospital of Wuhan University. The human specimen use was in line with the requirements of the Helsinki Declaration.

### Cell culture

Human colon cancer cell line (HT29, LOVO, HCT116, SW480) were purchased from Chinese Type Culture Centre (CTCC, China Wuhan). All cells were cultured in RPMI-1640 (HyClone, USA) containing 10% fetal bovine serum (Cell-Box, Australia) at 37 °C, 5% CO2.

#### SiRNA and plasmids transfection

PCMV3-SLC12A2 plasmids were purchased from Sino Biological (Beijing, China). SLC12A2 siRNA was synthesized by Ribobio (Guangzhou, China). SLC12A2 siRNA (siSLC12A2 #1: TGACCTTATTGATACCTTA, siSLC12A2 #2: GTAAGATCAGAGTATTCAT) was transfected into HT29 and LOVO cells using Lipofectamine 2000 (Invitrogen, USA). Scrambled siRNA (siNC) was used as a negative control. PCMV3-NEO1 plasmids (2.5 μg each well) were transfected to HCT116 and SW480 cells following the manufacturer’s protocol. Empty vector pCMV3 plasmids were used as control.

#### RNA preparation and quantitative real-time PCR (qPCR)

RNA was isolated from cells using Trizol reagent (ELK Biotechnology, China) according to the manufacturer's protocol. 1 μg of RNA was used to synthesise cDNA by cDNA Synthesis Kit (TOYOBO, Japan). cDNA was subsequently used for real-time fluorescence quantitative PCR (qPCR) using the ABI QuantStudio™6 Flex System (USA) and the Real-time fluorescence quantitative PCR (qPCR) was subsequently performed using ABI QuantStudio™6 Flex System (USA) and UltraSYBR Mixture (CWBIO, China). Gene primers: SLC12A2 (Forward 5′-CCTCTACACAAGCCCTGACTTAC-3′ and Reverse 5′-CGTGAGTTTGGAGCACCTGTCA-3′); GAPDH (Forward 5′-GTCTCCTCTGACTTCAACAGCG-3′ and Reverse 5′-ACCACCCTGTTGCTGTAGCCAA-3′). The relative mRNA expression levels were calculated using the 2^-△△Ct^ method.

#### Western blotting analysis

Proteins were collected from CRC cells and tissues using RIPA lysis buffer (EpiZyme, China), and protein concentrations were detected using a BCA kit (Beyotime, China). 30 μg of protein per group was loaded on a 10% SDS-PAGE gel, transferred to a PVDF membrane (Millipore, USA) and then closed with 5% skimmed milk. The blots were cut prior to hybridization with specific antibodies according to the protein molecular weight during blotting. Thereby, decreasing the amount of incubation solution used during the antibody incubation step, contributing to reduce the use of the costly antibodies. The membranes were incubated overnight at 4 °C in primary antibodies: SLC12A2 (13,884–1-AP, Proteintech, China), C-myc (5605S, CST, USA), Nanog (14,295–1-AP, Proteintech, China), CD44 (37259S, CST, USA), GAPDH (Servicebio, China). Finally, the cells were incubated with the corresponding secondary antibodies for 2 h and the bands were detected by enhanced chemiluminescence reagent (EpiZyme, China).

#### Cell growth assay

Cell proliferation was detected by Colony Formation and Cell Counting Kit 8 (CCK-8). For Colony Formation assay, 1500 colorectal cancer cells were seeded into 6-well plates with 2 ml of medium per well and cultured for 14 days. The number of colonies with more than 50 cells was counted after 14 days using 4% paraformaldehyde fixation and 0.1% crystal violet staining. As for CCK-8 assay, colorectal cancer cells were inoculated into 96-well plates at a number of 4000 per well and subsequently transfected with siRNA or plasmids for different groups. At specific time points, CCK-8 solution was added to the well plates and cultured for 2 h before detecting the OD450 by means of a microplate reader (BioTek ELx800, USA).

#### Statistical analysis

Differences in clinical characteristics between the high-risk and low-risk groups were analyzed using chi-square analysis. Student's *t*-test was used to compare normally distributed variables between the two groups. Two-tailed *p*-values < 0.05 were statistically significant.

### Ethics approval and consent to participate

This project was approved by the Ethics Committee of Zhongnan Hospital of Wuhan University (protocol #2023015 K). Informed consent was obtained from the participated patients.

## Results

### Identification and validation of a prognosis-related SLC gene signature in CRC

**Figure 1:**
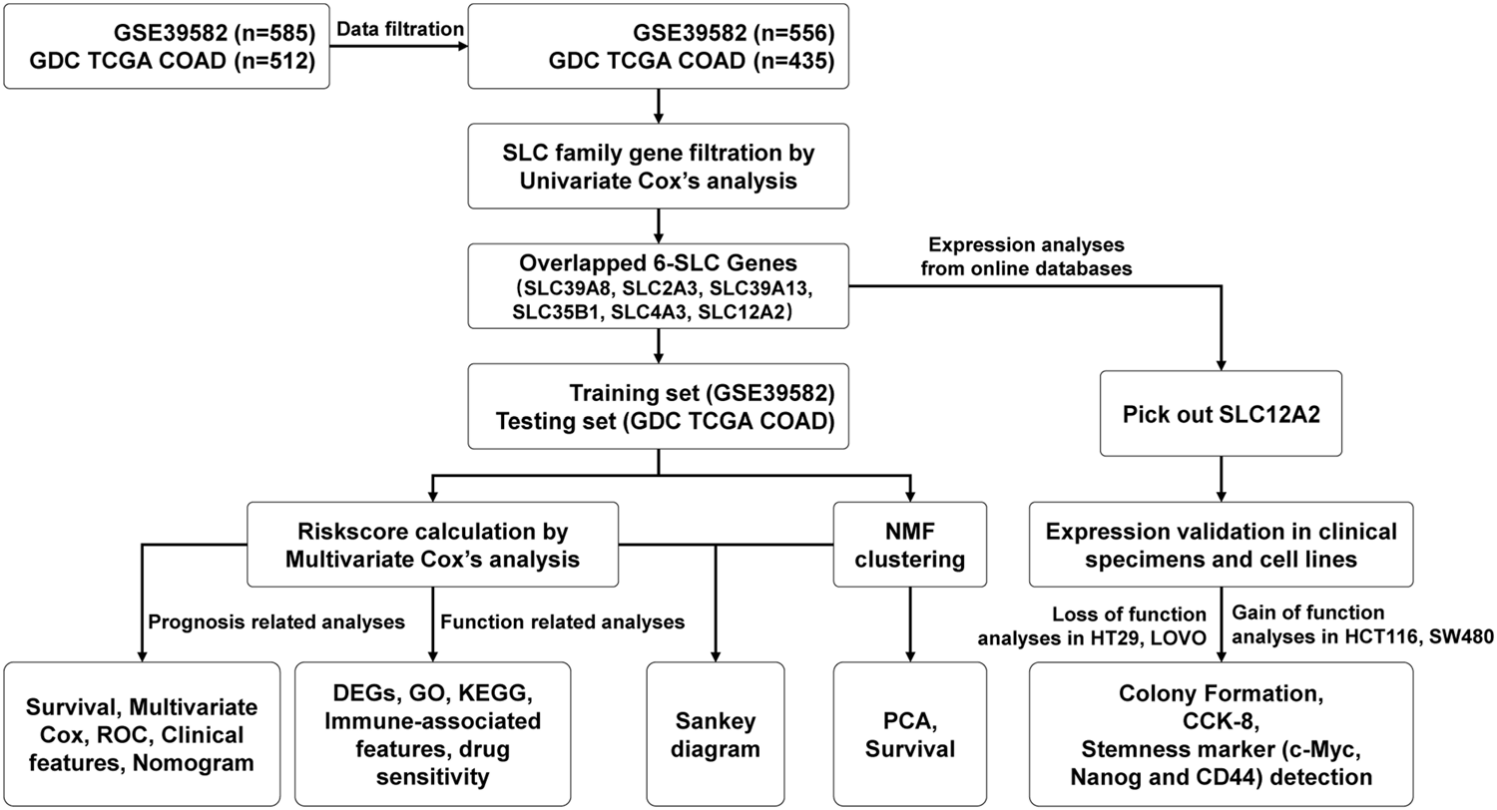
A flow chart of the study.

**Figure 2:**
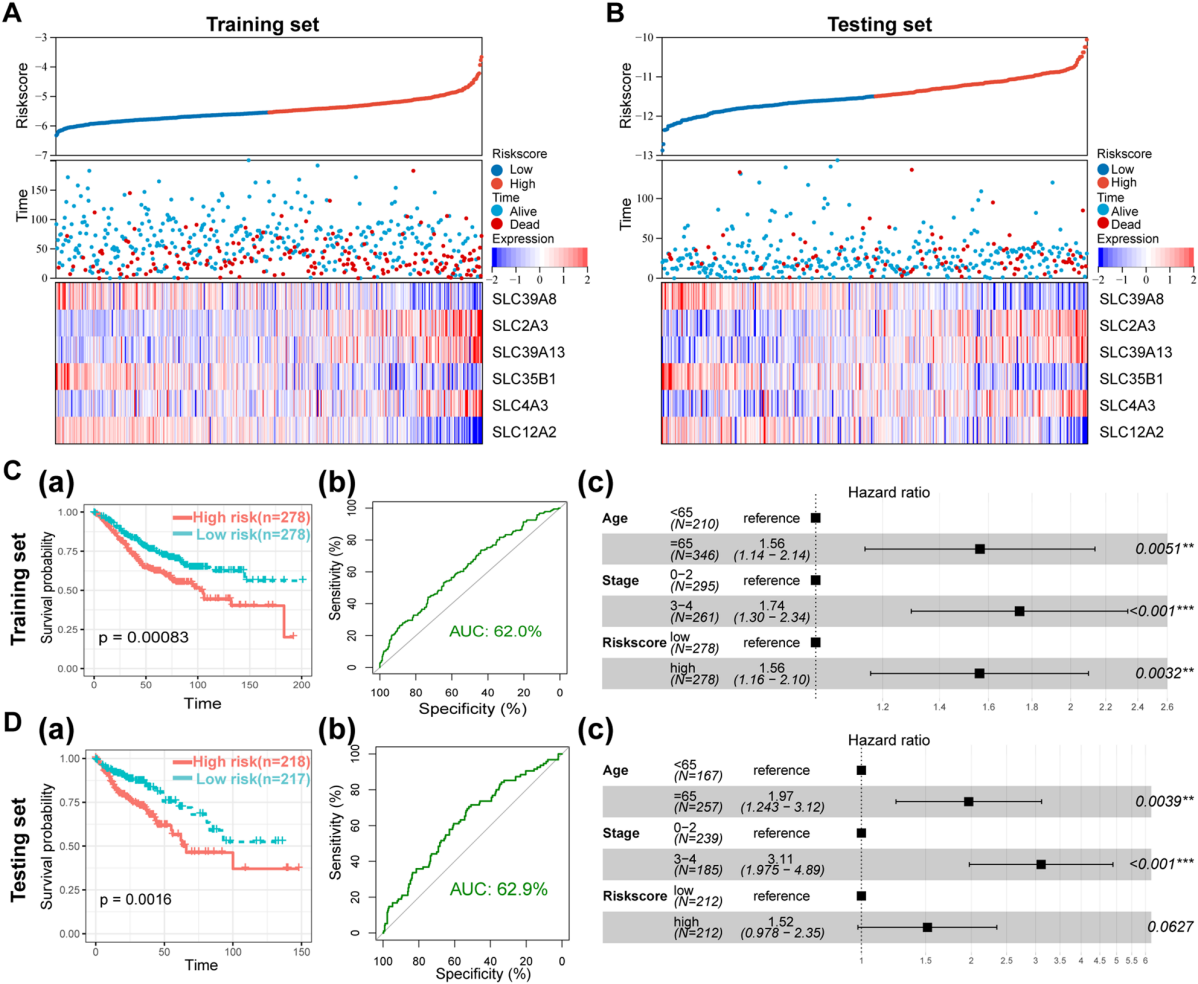
Construction of a prognosis-related SLC gene signature in CRC. **(A,B)** The distribution of risk scores and the correspondingly survival status of patients, expression abundance of the 6-SLC gene in the training set **(A)** and the testing set **(B)**. **(C)** Survival curves(a), ROC curves (b) and multivariate Cox regression analyses (c) in the training set. **(D)** Survival curves(a), ROC curves (b) and multivariate Cox regression analyses (c) in the testing set.

First of all, a flow chart was shown to introduce the design of this study (Fig. 1). The clinical characteristics of GSE39582 and TCGA COAD dataset had been shown in our previous work, and there was no significant difference in general features between two datasets^20^. After excluding the repeated genes and genes whose expression value was zero in any analyzed sample, the expression matrix of the SLC family genes combined with survival data in the two datasets was acquired for subsequent analysis. To screen out prognosis-related SLC genes, univariate cox proportional hazards model was conducted in GSE39582 and TCGA COAD dataset. 6 genes (SLC39A8, SLC2A3, SLC39A13, SLC35B1, SLC4A3, SLC12A2) with *p*-values < 0.05 in the two datasets were regarded as prognosis-related SLC genes. Of the 6 genes, SLC2A3, SLC39A13 and SLC4A3 were risk factors with hazard ratios (HRs) > 1 and SLC39A8, SLC35B1 and SLC12A2 were protective factors with HRs < 1 (Table S1). We also showed the survival curves of the 6 genes (Figure S1). To construct the prognosis-related SLC gene signature, we performed multivariate Cox regression analysis and calculated the risk score of each sample based on the expression and the regression coefficients of the 6 genes (Table S2). Then, patients were assigned to low-risk or high-risk group according to the median risk score in GSE39582 (training set) and TCGA COAD (testing set) dataset. The prognosis of CRC patients in the low-risk group was better than that in the high-risk group in training set and testing set. The heatmap was used to visualize the expression levels of the 6 genes in the low- and high-risk group (Fig. 2A,B).

To further identify the importance of the 6-SLC gene signature on the CRC patients’ prognosis, we performed survival, ROC and multivariate Cox regression analyses. In the training set, survival analysis revealed that patients with CRC in the low-risk group had a significantly higher survival probability compared to the patients in high-risk group (*p* = 0.00083) (Fig. 2C-a). ROC analysis showed that the area under the curve (AUC) was 0.62 for the training set (Fig. 2C-b). In addition, multivariate Cox regression analysis also confirmed the independent prognostic value of this signature (Fig. 2C-c). We further applied this signature into testing set and found the consistent results (Fig. 2D). Clearly, these data indicated the 6-SLC gene signature could contribute to prognosis prediction of CRC patients.

### Development of nomogram and calibration curves for CRC patients

**Figure 3:**
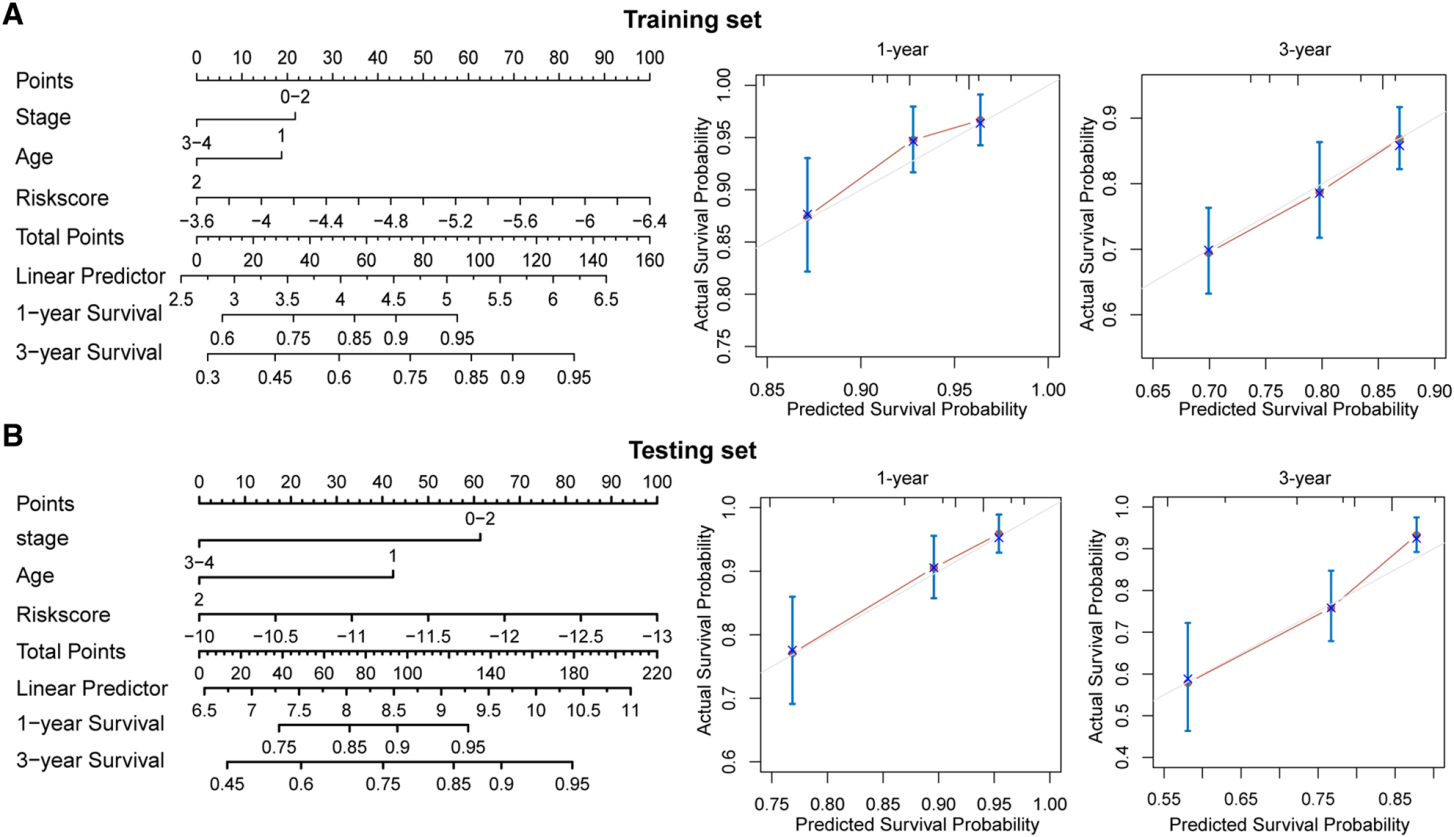
Development of nomogram and calibration curves for CRC patients. **(A,B)** The nomogram and calibration curves for CRC patient 1- and 3-year survival prediction in the training set **(A)** and the testing set **(B)**.

To accurately estimate the survival of individual CRC patients, we built a nomogram to assess 1- and 3-year survival probabilities based on staging, age, and risk scores in the training and test sets (Fig. 3A,B). Our results suggest that nomograms can be a useful model for prognostic assessment of CRC patients. The calibration curves showed that the predicted prognosis was generally consistent with the actual mortality rates at 1 and 3 years in both the training and test sets (Fig. 3A,B). These data suggest that the nomograms can accurately assess the survival of CRC patients.

### Clinical characteristics of CRC patients with different risk

**Figure 4:**
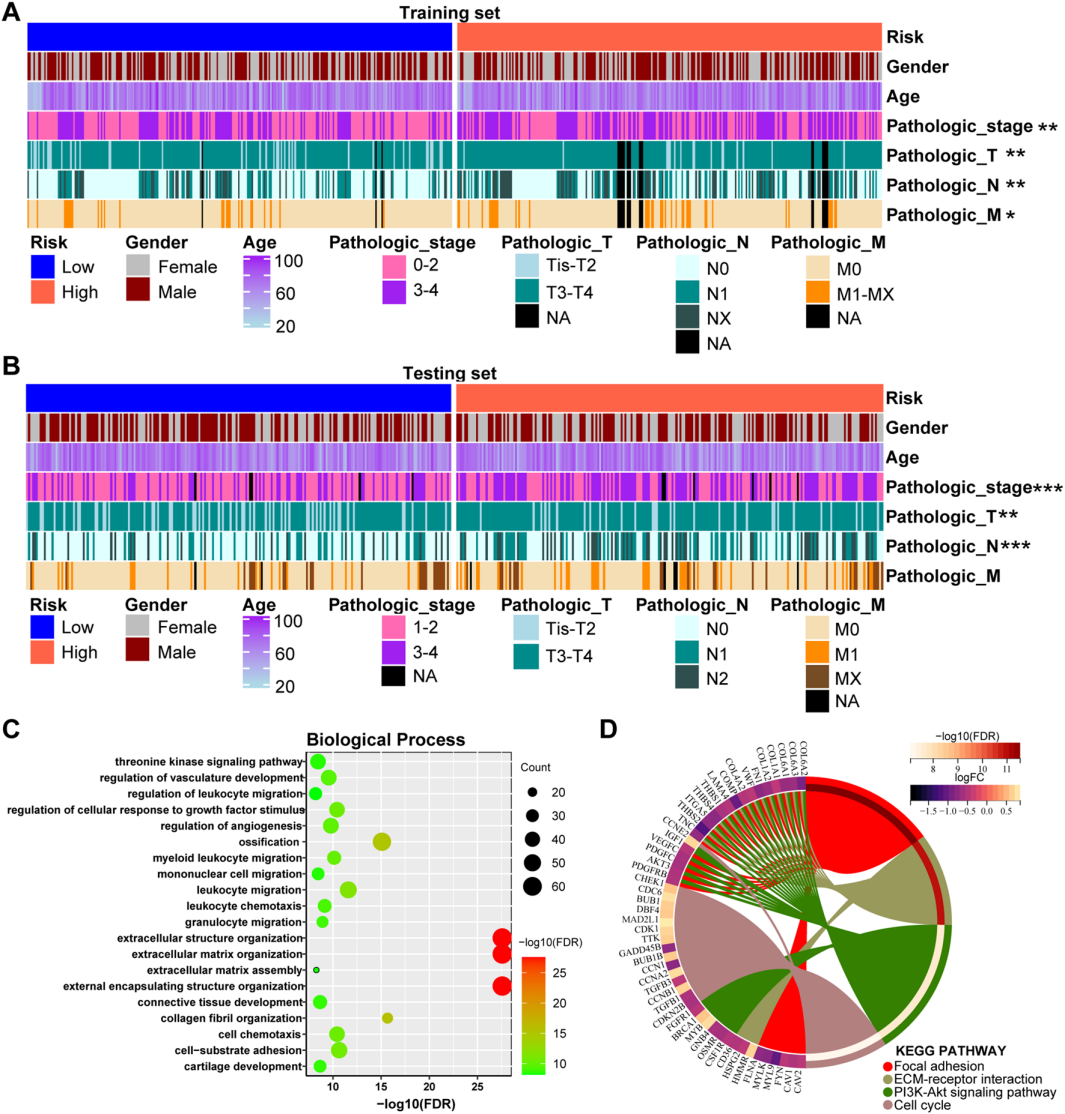
Clinical characteristics and functional enrichment analyses in different risk groups. **(A,B)** Heatmap of core clinical characteristics in different risk groups in the training set **(A)** and the testing set **(B)**. **(C)** GO analysis for biological process of identified DEGs in the training set. **(D)** KEGG analysis of identified DEGs in the training set. *P < 0.05, **P < 0.01, ***P < 0.001, ****P < 0.0001.

To better clarify the differences of CRC patients in low-risk and high-risk groups, the relationship with several clinical characteristics was studied by Chi-square test. The results in training set was shown in Table 1, which demonstrated that the proportion of patients in “TNM stage”, “T stage”, “N stage” and “M stage” were significantly different within different risk groups. Consistently, the differences of “TNM stage”, “T stage” and “N stage” within different risk groups of testing set also had significance. However, the *p*-value of “M stage” in testing set was exactly equal to 0.05 (Table 2). A heatmap was further used to more clearly visualize the correlation between different risk groups and clinical characteristics in training set and testing set (Fig. 4A,B). We also explored the correlation between risk groups and “TP53”, “KRAS”, “BRAF” mutant status in training set. The results showed no significant differences (Table 1). As for MSI status, it showed significant differences in testing set, which was inconsistent with that in training set (Tables 1, 2). Since clinicopathological stage was closely related to the prognosis of patients with CRC, the above results demonstrated the superior performance of the 6-SLC gene signature for prognosis prediction.

**Table 1:** Clinical characteristics of patients with different risk in training set.

Clinical characteristics		Total	Low risk	High risk	Chi-square	*P* value
		*n* = 556	*n*(%)	*n*(%)		
Gender	male	306	154(50.3)	152(49.7)	0.029	0.865
	female	250	124(49.6)	126(50.4)		
Age	< 65	210	102(48.6)	108(51.4)	0.275	0.600
	≥ 65	346	176(50.9)	170(49.1)		
TNM stage	0–2	295	166(56.3)	129(43.7)	9.886	0.002**
	3–4	261	112(42.9)	149(57.1)		
T stage	Tis-T2	57	41(71.9)	16(28.1)	10.859	0.001**
	T3-T4	479	234(48.9)	245(51.1)		
	*NA*	20	3(15.0)	17(85.0)		
N stage	N0	296	170(57.4)	126(42.6)	12.940	0.002**
	N1	131	64(48.9)	67(51.1)		
	NX	109	41(37.6)	68(62.4)		
	*NA*	20	3(15.0)	17(85.0)		
M stage	M0	473	250(52.9)	223(47.1)	3.861	0.049*
	M1-MX	63	25(39.7)	38(60.3)		
	*NA*	20	3(15.0)	17(85.0)		
TP53	M	188	90(47.9)	98(52.1)	0.397	0.529
	WT	156	80(51.3)	76(48.7)		
	*NA*	212	108(50.9)	104(49.1)		
KRAS	M	213	111(52.1)	102(47.9)	0.811	0.368
	WT	322	155(48.1)	167(51.9)		
	*NA*	21	12(57.1)	9(42.9)		
BRAF	M	49	24(49.0)	25(51.0)	0.004	0.950
	WT	453	224(49.4)	229(50.6)		
	*NA*	54	30(55.6)	24(44.4)		
MMR	dMMR	72	42(58.3)	30(41.7)	2.221	0.136
	pMMR	438	214(48.9)	224(51.1)		
	*NA*	46	22(47.8)	24(52.2)		

*P < 0.05, **P < 0.01, ***P < 0.001.

**Table 2:** Clinical characteristics of patients with distinct risk in testing set.

Clinical characteristics		Total	Low risk	High risk	Chi-square	*P* value
		*n* = 435	*n*(%)	*n*(%)		
Gender	male	233	123(52.8)	110(47.2)	1.693	0.193
	female	202	94(46.5)	108(53.5)		
Age	< 65	171	83(48.5)	88(51.5)	0.205	0.651
	≥ 65	264	134(50.8)	130(49.2)		
TNM stage	1–2	239	140(58.6)	99(41.4)	16.120	< 0.0001***
	3–4	185	72(38.9)	113(61.1)		
	*NA*	11	5(45.5)	6(54.5)		
T stage	Tis-T2	87	57(65.5)	30(34.5)	10.630	0.001**
	T3-T4	348	160(46.0)	188(54.0)		
N stage	N0	255	150(58.8)	105(41.2)	20.527	< 0.0001***
	N1	102	41(40.2)	61(59.8)		
	N2	78	26(33.3)	52(66.7)		
M stage	M0	320	170(53.1)	150(46.9)	6.000	0.050
	M1	61	22(36.1)	39(63.9)		
	MX	47	23(48.9)	24(51.1)		
	*NA*	7	2(28.6)	5(71.4)		
MSI status	MSS	269	128(47.6)	141(52.4)	7.047	0.03*
	MSI-L	79	34(43.0)	45(57.0)		
	MSI-H	73	46(63.0)	27(37.0)		
	*NA*	14	9(64.3)	5(35.7)		

*P < 0.05, **P < 0.01, ***P < 0.001.

### Functional enrichment analyses of DEGs in different risk groups

In order to gain insight into the molecular characteristics of low- and high-risk group, we screened the DEGs and conducted their functional enrichment analyses in training dataset. Under a threshold of Adjusted P value < 0.05 and |log2FC|> 0.5, a total of 707 DEGs were identified for the different risk groups. In detail, 536 DEGs were down-regulated while 171 genes were up-regulated (Table S3). GO analysis including biological process (BP), cellular component (CC) and molecular function (MF) showed that the above DEGs were mostly enriched in extracellular matrix (ECM) related functions (Fig. 4C, Figure S2). Moreover, KEGG analysis also exhibited that the pathways of focal adhesion, ECM-receptor interaction, PI3K-Akt and cell cycle were particularly prominent in these DEGs (Fig. 4D).

### Immune infiltration and cancer progression estimation in different risk groups

**Figure 5:**
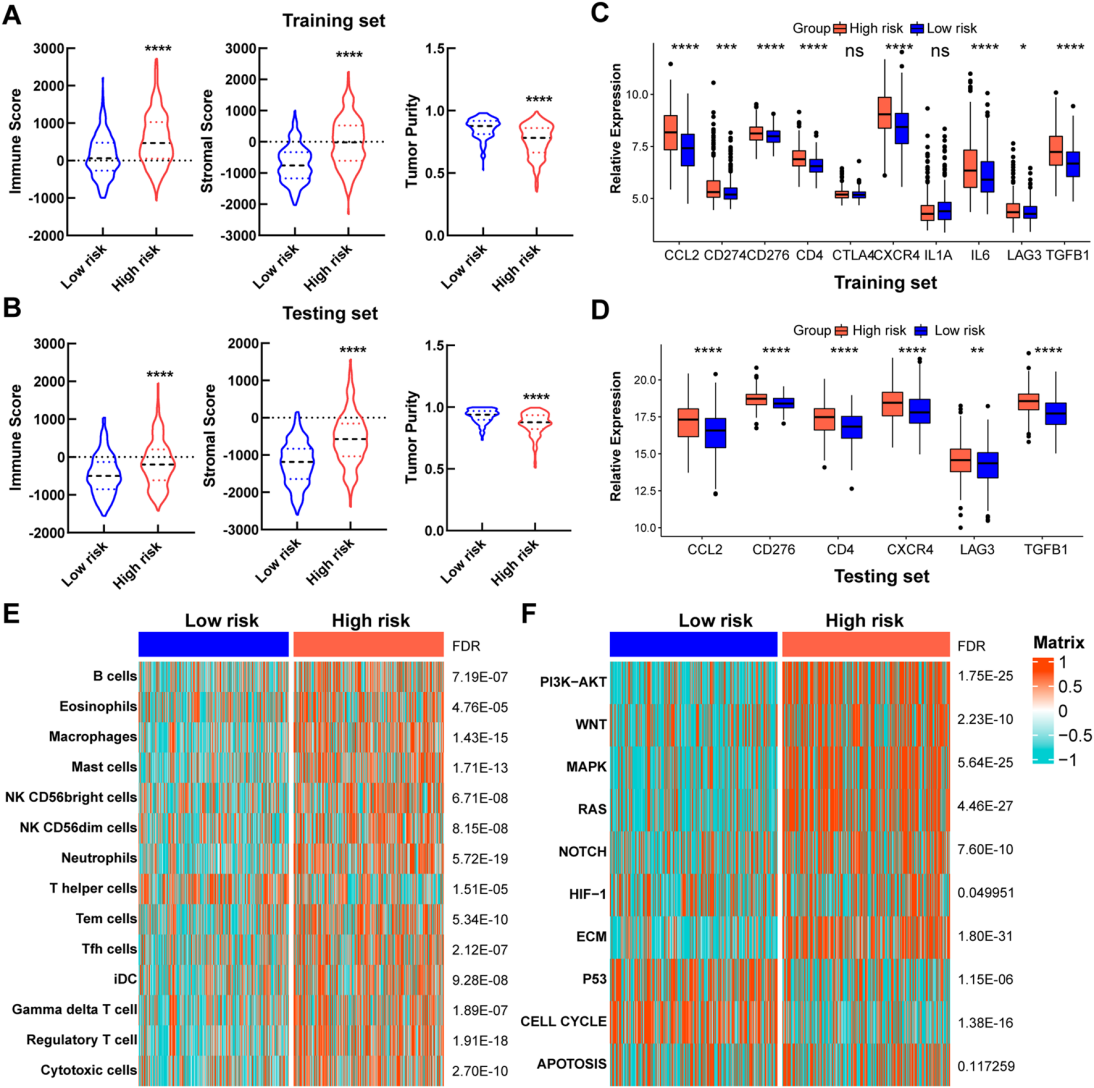
Immune infiltration and cancer progression estimation in different risk groups. **(A,B)** The violin plot of immune score, stromal score and tumor purity from ESTIMATE of two risk groups in the training set **(A)** and the testing set **(B)**. For violin plots, the three lines within the boxes represent the 25th percentile, median value and the 75th percentile, respectively. The bottom and top of the plots represent the min and max value. **(C,D)** Expression differences of several immune checkpoint genes between two risk groups in the training set **(C)** and the testing set **(D)**. **(E)** Heatmap describing the abundance of immune cell populations in two risk groups. **(F)** Heatmap describing the abundance of several CRC progression relevant pathways in two risk groups. *P < 0.05, **P < 0.01, ***P < 0.001, ****P < 0.0001, ns: no significance.

To evaluate the heterogeneity of tumors between low- and high-risk groups, we utilized the ESTIMATE algorithm to calculate stromal scores, immune scores, and tumor purity for both training and test sets. Results showed that the high-risk group had the higher immune score and stromal score, while the tumor purity of the high-risk group was lower than that of the low-risk group (Fig. 5A,B). The training set and the testing set exhibited the highly consistent results. We further investigated the expression of several immune checkpoints in the two risk groups. In training set, the expression of most immune checkpoints in the high-risk group were significantly higher than that in the low-risk group, except for CTLA4 and IL1A (Fig. 5C). Since CD274, CTLA4, IL1A and IL6 were not shown in the filtered expression matrix of the testing set, we analyzed the rest immune checkpoints, the results of which were coincided with that in training set (Fig. 5D). With the significant differences in immune score and immune checkpoints, immune infiltration was investigated to characterize their immunologic landscape. Based on a signature of 17 immune cell type (Table S4), immune cell infiltration was analyzed by GSEA in training set. The heatmap showed that immune cells were significantly enriched in the high-risk group, which was consistent with the result that the group had higher immune scores (Fig. 5E).

For further investigation, several CRC progression relevant pathways were also evaluated in training set (Table S5). Results exhibited that the high-risk group displayed higher expression for PI3K-AKT, WNT, MAPK, RAS, NOTCH, HIF-1 and ECM pathways, while the low-risk group was especially enriched with P53 and CELL CYCLE pathways. APOTOSIS pathway had no significance between two groups (Fig. 5F).

### Therapeutic drug sensitivity prediction of CRC in different risk groups

**Figure 6:**
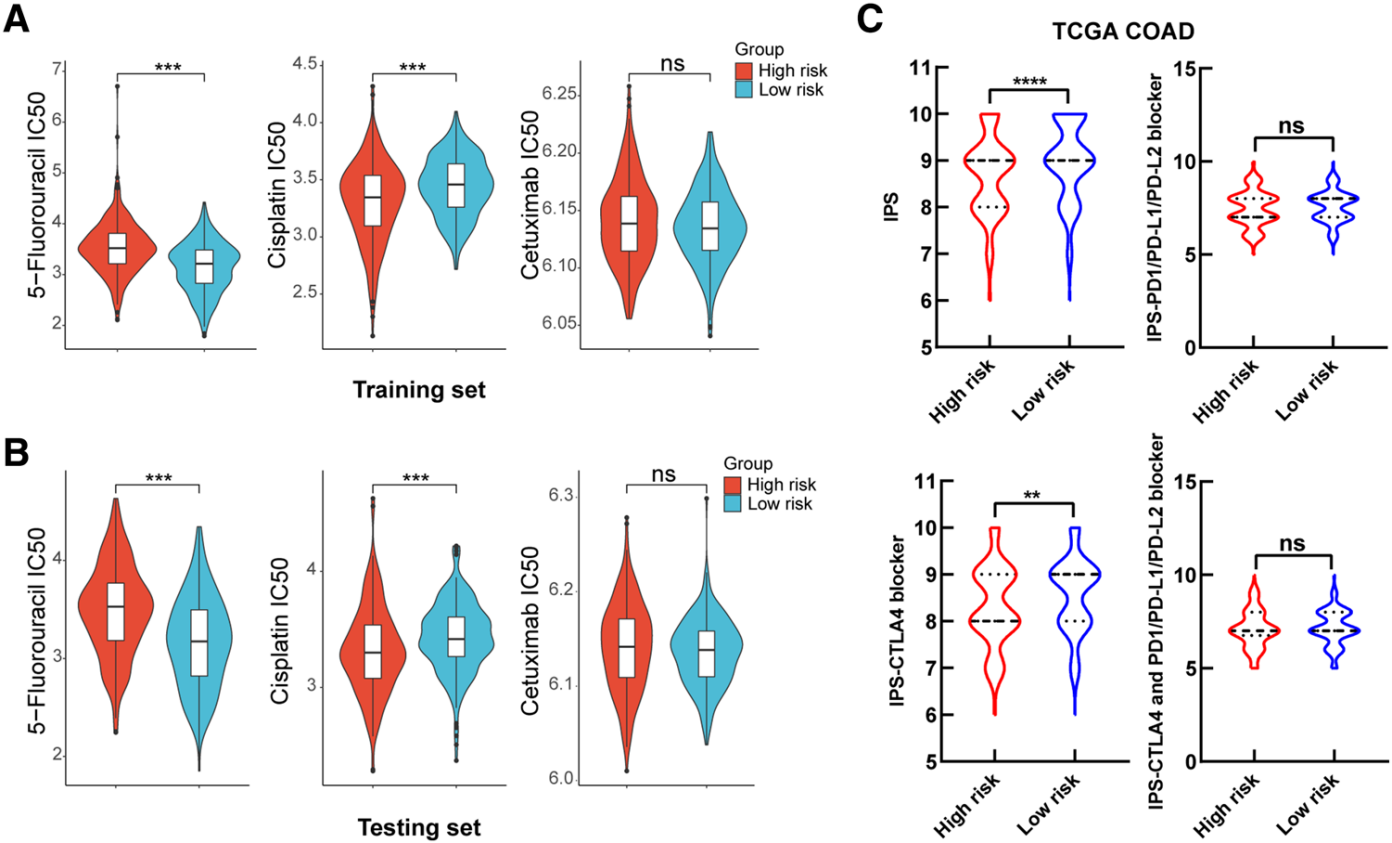
Drug sensitivity prediction to different therapies in different risk groups. **(A,B)** IC50 was calculated for 5-Fluorouracil, Cisplatin and Cetuximab in two risk groups in the training set **(A)** and the testing set **(B)**. **(C)** Immunophenoscore (IPS) difference of CRC with treatment of CTLA-4 or (and) PD1/PD-L1/PD-L2 blocker between the high- and low-risk groups. **P < 0.01, ***P < 0.001, ****P < 0.0001, ns: no significance.

Due to the high heterogeneity of CRC, it’s important to choose a therapeutic drug with high sensitivity. Therefore, we explored the relationship between the risk score and clinical response to different therapeutic drugs. Based on the Cancer Genome Project (CGP) database, we screened two commonly used chemotherapy drugs (5-Fluorouracil and Cisplatin) and one targeted drug (Cetuximab) for CRC treatment to calculate IC50. The results showed that, both in training set and testing set, CRC patients in the low-risk group were more sensitive to the treatment of 5-Fluorouracil, while patients in the high-risk group had a favorable response to the treatment of Cisplatin. However, there was no significant differences for the treatment of Cetuximab (Fig. 6A,B). Besides, the cancer immunome atlas (TCIA) is a database that provides comprehensive immunogenomic analyses based on the TCGA. Here, we used the TCIA database to evaluate the immunotherapy response of CRC patients with different risk scores through the immunophenoscore (IPS). The results revealed that the total IPS and IPS for CTLA-4 blocker in the low-risk group were significantly higher than that in the high-risk group (Fig. 6C), which strongly predicted that CRC patients with lower risk scores would have better immunotherapy response, especially for CTLA-4 blocker.

### Molecular subtyping based on the prognosis-related SLC genes for CRC patients

**Figure 7:**
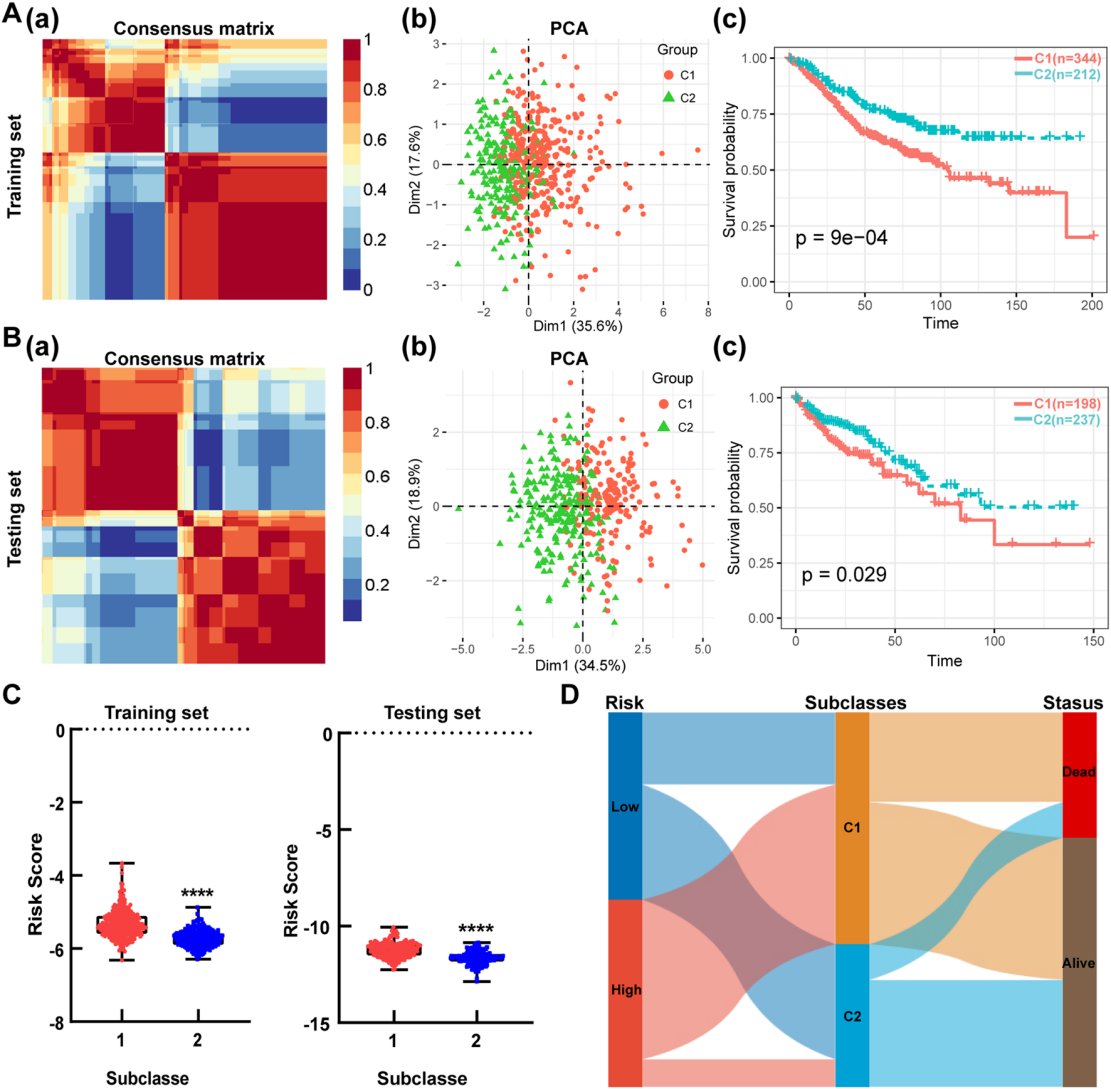
Identification of subclasses based on the screened SLC genes for CRC patients. **(A,B)** Two distinct subclasses (C1, C2) was identified by NMF clustering in the training set **(A)** and the testing set **(B)**. The consensus map (a), PCA (b) and survival analysis (c) of two subclasses were shown. **(C)** The risk scores of two subclasses in the training set and testing set. **(D)** Sankey diagram for the two risk groups and two subtypes. ****P < 0.0001.

Recently, molecular subtyping was broadly applied to reveal the tumor heterogeneity. To more comprehensively identify the importance of the 6 prognosis-related SLC genes we selected, NMF clustering was executed. The consensus map showed that patients with CRC were classified into two distinct clusters in the training set (Fig. 7A-a). There were 344 samples in the cluster 1 (C1) and 212 in the C2. To access the subclasses’ assignments, we performed PCA. Data showed that the two clusters were distributed in different side of the two-dimensional coordinate systems (Fig. 7A-b). Survival analysis showed that the survival probability of patients in C1 was significantly lower than that of patients in C2 (Fig. 7A-c). Furthermore, a similar NMF consensus clustering was acquired in the testing set (Fig. 7B-a). Consistently, two subclasses were identified, which also manifested the same distribution as that in training set by PCA (Fig. 7B-b). In the testing set, patients in C1 also had a significantly lower survival probability compared to the patients in C2 (Fig. 7B-c). The correlation analysis of clinical characteristics showed that the proportion of patients in “TNM stage”, “T stage” and “N stage” were significantly different within distinct subclasses (Tables 3 and 4). We then calculated the risk scores in different subclasses in the both training set and testing set. Data showed that C1 had higher risk scores than C2 (Fig. 7C). The Sankey showed that patients in the high-risk group and C1 subtype had a worse prognosis (Fig. 7D). These data sufficiently demonstrated that the 6-SLC genes could be applied to prognosis prediction.

**Table 3:** Clinical characteristics of patients with distinct classification in training set.

Clinical characteristics		Total	C1	C2	Chi-square	*P* value
		*n* = 556	*n*(%)	*n*(%)		
Gender	male	306	196(64.1)	110(35.9)	1.373	0.241
	female	250	148(59.2)	102(40.8)		
Age	< 65	210	129(61.4)	81(38.6)	0.028	0.867
	≥ 65	346	215(62.1)	131(37.9)		
TNM stage	0–2	295	163(55.3)	132(44.7)	11.661	0.001**
	3–4	261	181(69.3)	80(30.7)		
T stage	Tis-T2	57	24(42.1)	33(57.9)	10.426	0.001**
	T3-T4	479	307(64.1)	172(35.9)		
	*NA*	20	13(65.0)	7(35.0)		
N stage	N0	296	165(55.7)	131(44.3)	15.385	< 0.0001***
	N1	131	82(62.6)	49(37.4)		
	NX	109	84(77.1)	25(22.9)		
	*NA*	20	13(65.0)	7(35.0)		
M stage	M0	473	285(60.3)	188(39.7)	3.834	0.050
	M1-MX	63	46(73.0)	17(27.0)		
	*NA*	20	13(65.0)	7(35.0)		
TP53	M	188	110(58.5)	78(41.5)	0.479	0.489
	WT	156	97(62.2)	59(37.8)		
	*NA*	212	137(64.6)	75(35.4)		
KRAS	M	213	127(59.6)	86(40.4)	0.889	0.346
	WT	322	205(63.7)	117(36.3)		
	*NA*	21	12(57.1)	9(42.9)		
BRAF	M	49	41(83.7)	8(16.3)	9.663	0.002**
	WT	453	277(61.1)	176(38.9)		
	*NA*	54	26(48.1)	28(51.9)		
MMR	dMMR	72	53(73.6)	19(26.4)	5.297	0.021*
	pMMR	438	260(59.4)	178(40.6)		
	*NA*	46	31(67.4)	15(32.6)		

*P < 0.05, **P < 0.01, ***P < 0.001.

**Table 4:** Clinical characteristics of patients with distinct classification in testing set.

Clinical characteristics		Total	C1	C2	Chi-square	*P* value
		*n* = 435	*n*(%)	*n*(%)		
Gender	male	233	105(45.1)	128(54.9)	0.041	0.839
	female	202	93(46.0)	109(54.0)		
Age	< 65	171	81(47.4)	90(52.6)	0.389	0.533
	≥ 65	264	117(44.3)	147(55.7)		
TNM stage	1–2	239	96(40.2)	143(59.8)	8.090	0.004**
	3–4	185	100(54.1)	85(45.9)		
	*NA*	11	2(18.2)	9(81.8)		
T stage	Tis-T2	87	28(32.2)	59(67.8)	7.796	0.005**
	T3-T4	348	170(48.9)	178(51.1)		
N stage	N0	255	101(39.6)	154(60.4)	8.763	0.013*
	N1	102	54(52.9)	48(47.1)		
	N2	78	43(55.1)	35(44.9)		
M stage	M0	320	138(43.1)	182(56.9)	4.341	0.114
	M1	61	35(57.4)	26(42.6)		
	MX	47	20(42.6)	27(57.4)		
	*NA*	7	5(71.4)	2(28.6)		
MSI status	MSS	269	118(43.9)	151(56.1)	0.931	0.628
	MSI-L	79	38(48.1)	41(51.9)		
	MSI-H	73	36(49.3)	37(50.7)		
	*NA*	14	6(42.9)	8(57.1)		

*P < 0.05, **P < 0.01, ***P < 0.001.

### Expression analysis of the prognosis-related SLC genes in CRC

**Figure 8:**
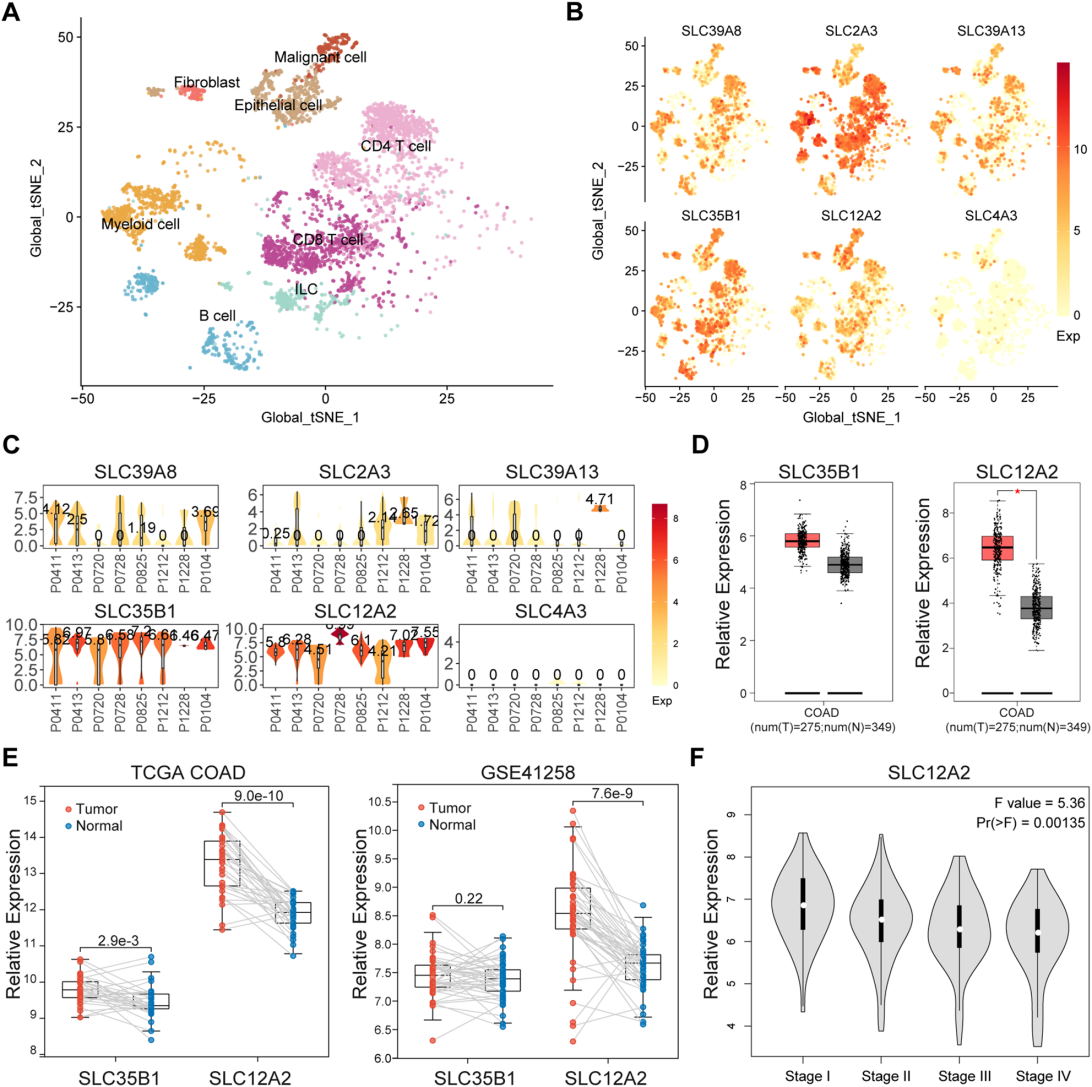
Expression analysis of the 6 prognosis-related SLC genes in CRC. **(A)** A tSNE map of global cell clusters from 8 CRC samples. **(B)** Expression distribution in different cell clusters of the 6 SLC genes. **(C)** Expression levels in malignant cells from 8 CRC samples of the 6 SLC genes. **(D)** Expression levels of SLC35B5 and SLC12A2 in CRC tumor tissues and normal tissues from GEPIA. **(E)** Expression levels of SLC35B5 and SLC12A2 in paired tumor tissues and adjacent normal tissues in TCGA COAD and GSE41258. **(F)** Expression levels of SLC12A2 in the different tumor stage from GEPIA. *P < 0.05.

To better realize the 6 prognosis-related SLC genes, their expression distribution in different cell clusters of 8 CRC samples was shown by single cell RNA-sequencing data. A t-Distributed stochastic neighbor embedding (tSNE) map demonstrated that of the 6 prognosis-related SLC genes, the expression of SLC35B5 and SLC12A2 were more enriched in malignant cells (Fig. 8A,B). The expression levels of the 6-SLC genes in malignant cells were further shown by the violin plots, from which we could clearly find that SLC35B5 and SLC12A2 were widely and highly expressed in each CRC sample (Fig. 8C). According to Gene Expression Profiling Interactive Analysis (GEPIA) database (https://gepia.cancer-pku.cn/), we found that SLC35B5 and SLC12A2 were both upregulated in tumor tissues compared with normal tissues of CRC. However, the comparison of SLC35B1 had no significance (Fig. 8D). Furthermore, we extracted expression data of SLC35B5 and SLC12A2 from paired tumor tissues and adjacent normal tissues in TCGA COAD and GSE41258. Results showed that SLC12A2 was consistently and significantly upregulated in tumor tissues of both datasets (Fig. 8E). We also found that the expression of SLC12A2 was decreased with the progression of CRC from GEPIA (Fig. 8F). The above data revealed that SLC12A2 was specially enriched in malignant cells of CRC and obviously increased in tumor tissues compared to normal tissues.

### SLC12A2 regulated cell proliferation and stemness in CRC cells

**Figure 9:**
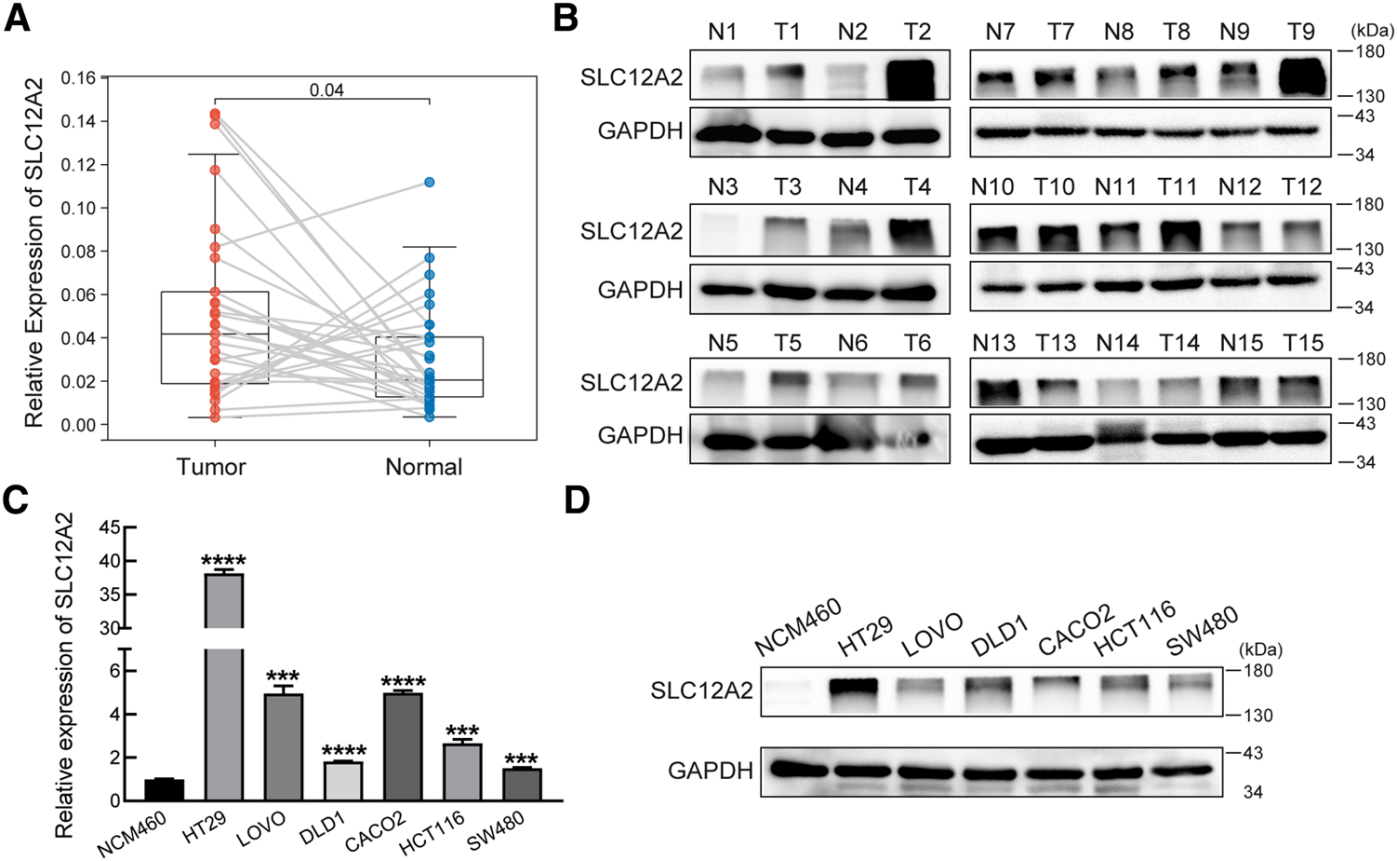
Validation the expression of SLC12A2 in CRC. **(A)** The expression of SLC12A2 in 29 pairs of CRC tumor tissues and adjacent non-tumor tissues was tested by real-time PCR. **(B)** The expression of SLC12A2 in 15 pairs of CRC tumor tissues and adjacent non-tumor tissues was tested by western blotting. **(C,D)** The expression of SLC12A2 in 6 CRC cell lines and normal colon cells were tested by real-time PCR **(C)** and western blotting **(D)**. The western blot images in this figure were cropped from full blots and original full blots were presented in Supplementary Fig. 3. Three dependent experiments were performed with similar results. ***P < 0.001, ****P < 0.0001.

**Figure 10:**
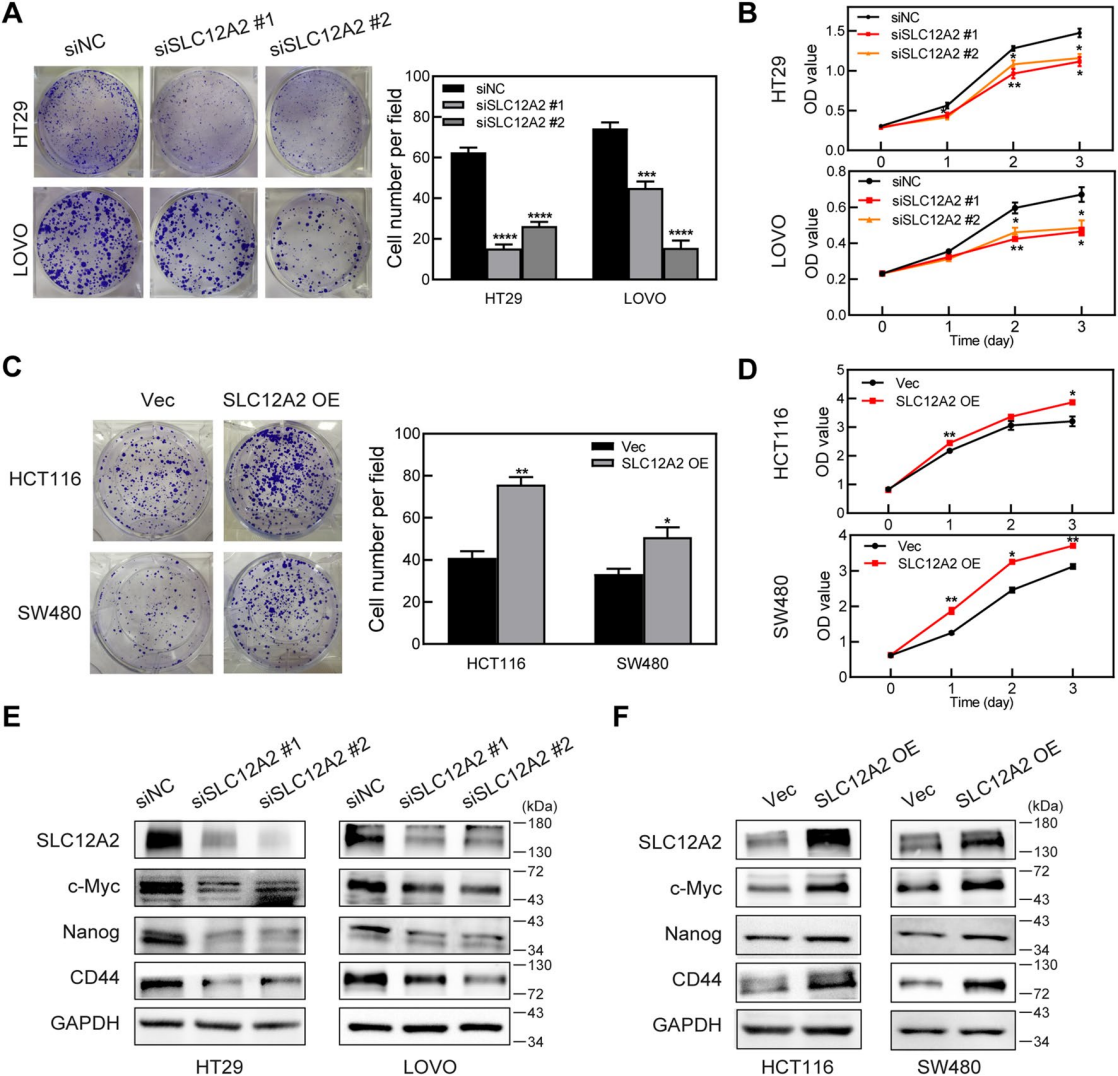
Identification the role of SLC12A2 in CRC cells. **(A,B)** Colony formation assay **(A)** and CCK-8 assay **(B)** in HT29 and LOVO cells transfected with SLC12A2 siRNAs (siSLC12A2 #1, #2) or control siRNAs (siNC). **(C,D)** Colony formation assay **(C)** and CCK-8 assay **(D)** in HCT116 and SW480 cells transfected with pCMV3-SLC12A2 plasmids (SLC12A2 OE) or control empty vectors (Vec). **(E)** The expression of SLC12A2, c-Myc, Nanog and CD44 were tested by western blotting in HT29 and LOVO cells transfected with SLC12A2 siRNAs (siSLC12A2 #1, #2) or control siRNAs (siNC). **(F)** The expression of SLC12A2, c-Myc, Nanog and CD44 were tested by western blotting in HCT116 and SW480 cells transfected with pCMV3-SLC12A2 plasmids (SLC12A2 OE) or control empty vectors (Vec). The western blot images in this figure were cropped from full blots and original full blots were presented in Supplementary Fig. S3. Three dependent experiments were performed with similar results. Data shown above were presented as the means ± s.e.m., *n* = 3; *P < 0.05, **P < 0.01, ***P < 0.001, ****P < 0.0001.

The above results demonstrated that SLC12A2 expression was significantly up-regulated in human CRC tissues. For validation, we collected 29 pairs of tumor tissues and adjacent non-tumor tissues in Zhongnan Hospital of Wuhan University. The results confirmed that SLC12A2 was up-regulated in CRC tumor tissues when compared with non-tumor tissues, both at mRNA level (*p* = 0.04,

*n* = 29) (Fig. 9A) and protein level (*n* = 15) (Fig. 9B). Then, SLC12A2 expression level in several kinds of CRC cells was examined. Compared to normal colon cell NCM460, SLC12A2 showed higher expression in CRC cells (Fig. 9C,D). In order to further investigate the biological role of SLC12A2 in CRC cells, two specific SLC12A2 siRNAs (siSLC12A2 #1, #2) were used to knockdown SLC12A2 expression in HT29 and LOVO cells while pCMV3-SLC12A2 plasmids were used to overexpress SLC12A2 expression in HCT116 and SW480 cells. The results of colony formation and CCK-8 assays showed that cell proliferation was distinctly inhibited in SLC12A2 knockdown cells compared with the control cells (Fig. 10A,B). One the other hand, SLC12A2 overexpression induced cell proliferation in HCT116 and SW480 cells (Fig. 10C,D). What’s more, western blotting assays showed that knockdown of SLC12A2 down-regulated the expression of the stemness marker c-Myc, Nanog and CD44 while overexpression of SLC12A2 showed the contrary results (Fig. 10E,F). The above data partly indicated that SLC12A2 was involved in CRC progression by promoting cell stemness.

## Discussion

As the largest transporter family, SLC membrane transporters had been used to construct the prognosis related signature in lung adenocarcinoma^21^, osteosarcoma^22^, hepatocellular carcinoma^23^, renal cell carcinoma^24^ and pancreatic ductal adenocarcinoma^25^. Here, we devoted to reveal a prognosis related SLC gene signature in CRC, which has not been reported before. Our study identified and validated a 6-SLC gene (SLC39A8, SLC2A3, SLC39A13, SLC35B1, SLC4A3, SLC12A2) signature that could predict prognosis of CRC patients. We classified the samples in the training and testing datasets into low-risk or high-risk group based on the expression level of the 6-SLC genes. Different risk group was associated with different prognosis, clinical traits, molecular features, functions, and immune cell fractions.

In detail, results showed that CRC patients in the high-risk group had the poorer prognosis. Clinical feature analyses showed that most samples in the high-risk group were in advanced pathological stage. CRC progression signatures, such as PI3K-AKT, WNT, MAPK, RAS, NOTCH, HIF-1 and ECM were also enriched in the high-risk group. Moreover, tumor microenvironment relevant estimation demonstrated that the high-risk group had the higher immune score, stromal score and the lower tumor purity. These data suggested that tumors in the high-risk group were of high heterogeneity and might be refractory. Our opinion was also consistent with the results that the high-risk group had the worse prognosis in both training and testing sets. The high-risk group was filled with a variety of immune cells, and there was a high expression of immune checkpoint genes in the high-risk group, particularly CD274 (also known as PD-L1), CXCR4,CD276, CD4, IL6, LAG3, CCL2, and TGFB1, suggesting that PD-L1 antibodies (e.g., Nivolumab, Durvalumab) and other promising checkpoint inhibitors may be sensitive^26^. Additionally, our analyses revealed that CRC patients in the low-risk group had a favorable response to the treatment of 5-Fluorouracil and CTLA-4 blocker, which suggested that the 6-SLC gene signature could also be well applied to choose individualized therapies for CRC.

On the other hand, we also developed a molecular subtyping based on the prognosis-related SLC genes for CRC patients. Two distinct subclasses were identified, which showed different prognosis, clinical traits and risk scores. We also showed that patients in the high-risk group and C1 subtype had a worse prognosis.

Furthermore, a detailed analysis of the 6 prognosis-related SLC genes was implemented. We found that SLC35B5 and SLC12A2 were more expressed in malignant cells of CRC compared with other selected prognosis-related SLC genes. Hence, we evaluated the expression differences of SLC35B5 and SLC12A2 in tumor and normal tissues of CRC. In multiple datasets, we found that SLC12A2 was steadily upregulated in tumor tissues compared with not only over all normal tissues but also paired normal tissues of CRC, which was in consistent with others^27^. What’s more, a recent study showed a specific SLC12A2 immunohistochemical staining pattern in precancerous and cancerous colonic UC lesions, which could be helpful for diagnosing dysplasia and cancer in UC and non-UC patients^28^. Thus, we decided to explore the role of SLC12A2 in CRC, which has not been reported before.

The gene *SLC12A2* encodes the Na/K/2Cl cotransporter NKCC1, which mainly regulates intracellular ion concentrations and cell volume and plays important functions in neurons and epithelial cells^29^. So far, the role of SLC12A2 in different types of tumors has not been well defined. A study revealed that NKCC1/*SLC12A2* was highly expressed in Glioblastomas and it promoted EMT-like process via RhoA and Rac1 signaling pathways^30^. Moreover, inhibition of the NKCC1 could reduce glioma invasion^31^. Detailly, NKCC1 modulated migration of glioma cells by two distinct mechanisms, one was to regulate focal adhesion dynamics and cell contractility; the other was to regulate cell volume through ion transport^32^. Recently, a study showed that blockade of NKCC1 could increase Temozolomide (a conventional chemotherapy drug) induced glioma apoptosis and reduced astrogliosis, which presented the potential therapeutic effect of NKCC1^33^. Besides glioma, it was reported that high expression of NKCC1 predicted poor clinical outcomes for lung adenocarcinoma patients and an EGFR-mutated subgroup^34^. In our study, we proved that SLC12A2 could promote cell growth and stemness in CRC cells.

Here, we presented a pioneer work for identifying a prognosis-related SLC gene signature in CRC. However, we have to mention some defects. Firstly, more datasets are needed to verify our signature. Then, the validation of our signature in clinical samples is necessary. Moreover, our univariate cox proportional hazards model showed that SLC12A2 was a protective factor in CRC while our basic experiments showed that SLC12A2 promoted cancer progression in CRC cells, this inconsistency needs to be explored further. Besides, our data showed that the expression of SLC12A2 was decreased in a stage-dependent manner, the causes also remain unclear. We thought the expression of SLC12A2 might be influenced by the tumor microenvironment, the different therapeutic drugs and so on, which needs to be studied by in vitro and in vivo experiments further.

## Conclusions

Overall, our works deepened the understanding of SLC family genes in CRC, and provided a 6-SLC gene signature for prognosis prediction of CRC patients. At the same time, we have tentatively revealed the role of SLC12A2 in CRC, which may serve as a potential therapeutic target.

### Supplementary Information

Supplementary Legends.

Supplementary Figure S1.

Supplementary Figure S2.

Supplementary Figure S3.

Supplementary Tables.

## Data Availability

The TCGA data were extracted from GDC data portal (https://portal.gdc.cancer.gov/). The GSE39582 gene expression profiles were downloaded from GEO (https://www.ncbi.nlm.nih.gov/geo).

## Abbreviations

SLC: Solute carrier
CRC: Colorectal cancer
DEG: Differentially expressed gene
NMF: Non-negative matrix factorization
PCA: Principal components analysis
GO: Gene ontology
KEGG: Kyoto encyclopedia of genes and genomes
GSVA: Gene set variation analysis
CCK-8: Cell counting kit 8
tSNE: T-distributed stochastic neighbor embedding
GEPIA: Gene expression profiling interactive analysis
