# Supplementary material for: Comprehensive analyses of solute carrier family members identify SLC12A2 as a novel therapy target for colorectal cancer
Source: Sci Rep. 2024 Feb 23;14:4459. doi: 10.1038/s41598-024-55048-y (PMC10891168; doi:10.1038/s41598-024-55048-y)
Supplement: Supplementary file 1 — Supplementary Legends. [file 41598_2024_55048_MOESM1_ESM.pdf]

Supplementary figure legends

Figure S1. The survival curves of the 6 prognosis-related SLC genes. (A, B) The survival curves of the 6-SLC gene in the training set **(A)** and the testing set **(B)**.

Figure S2. GO analyses in different risk groups. (A, B) GO analysis for cellular component **(A)** and molecular function **(B)** of identified DEGs in the training set.
