# Supplementary figures and images for: Comprehensive analyses of solute carrier family members identify SLC12A2 as a novel therapy target for colorectal cancer
Source: Sci Rep. 2024 Feb 23;14:4459. doi: 10.1038/s41598-024-55048-y (PMC10891168; doi:10.1038/s41598-024-55048-y)

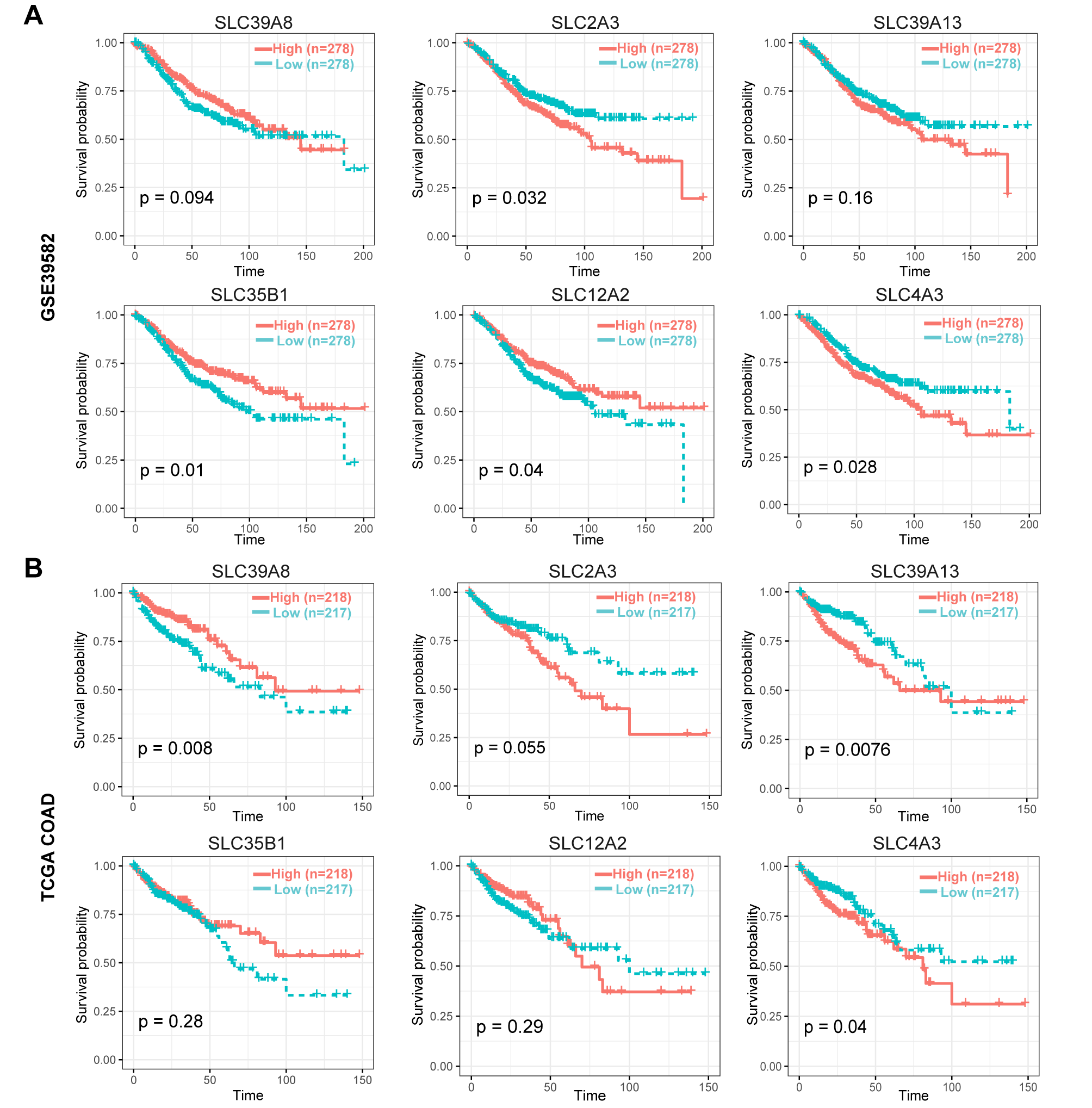

Supplement: Supplementary file 2 — Supplementary Figure S1. [file 41598_2024_55048_MOESM2_ESM.tif]

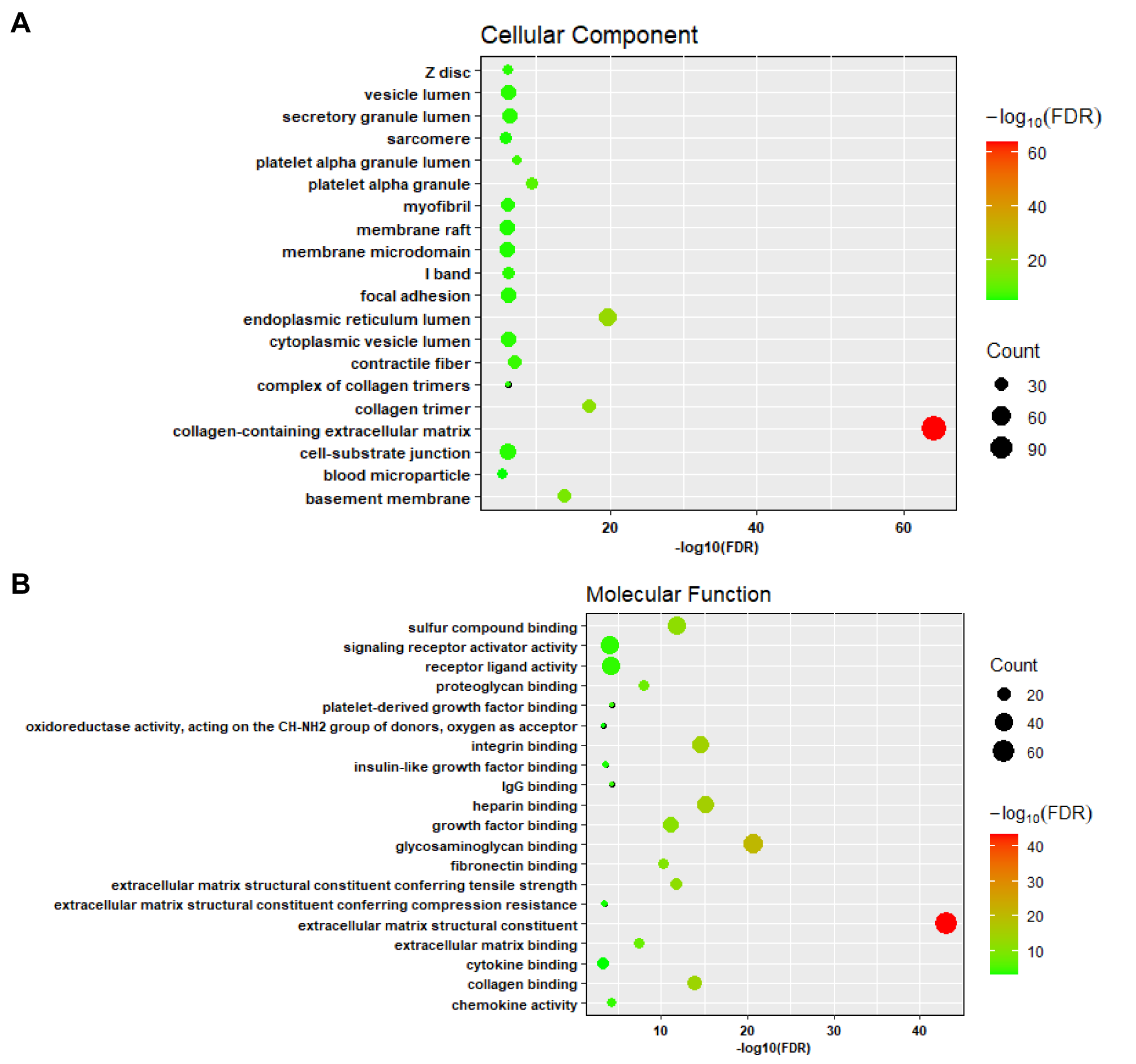

Supplement: Supplementary file 3 — Supplementary Figure S2. [file 41598_2024_55048_MOESM3_ESM.tif]
