# Supplementary material for: Comprehensive analyses of solute carrier family members identify SLC12A2 as a novel therapy target for colorectal cancer
Source: Sci Rep. 2024 Feb 23;14:4459. doi: 10.1038/s41598-024-55048-y (PMC10891168; doi:10.1038/s41598-024-55048-y)
Supplement: Supplementary file 4 — Supplementary Figure S3. [file 41598_2024_55048_MOESM4_ESM.pdf]

Supplementary Figure 3. The original images of blots. The blots were cut prior to hybridization with specific antibodies according to the protein molecular weight during blotting. Thereby, decreasing the amount of incubation solution used during the antibody incubation step, contributing to reduce the use of the costly antibodies.

Figure 9B

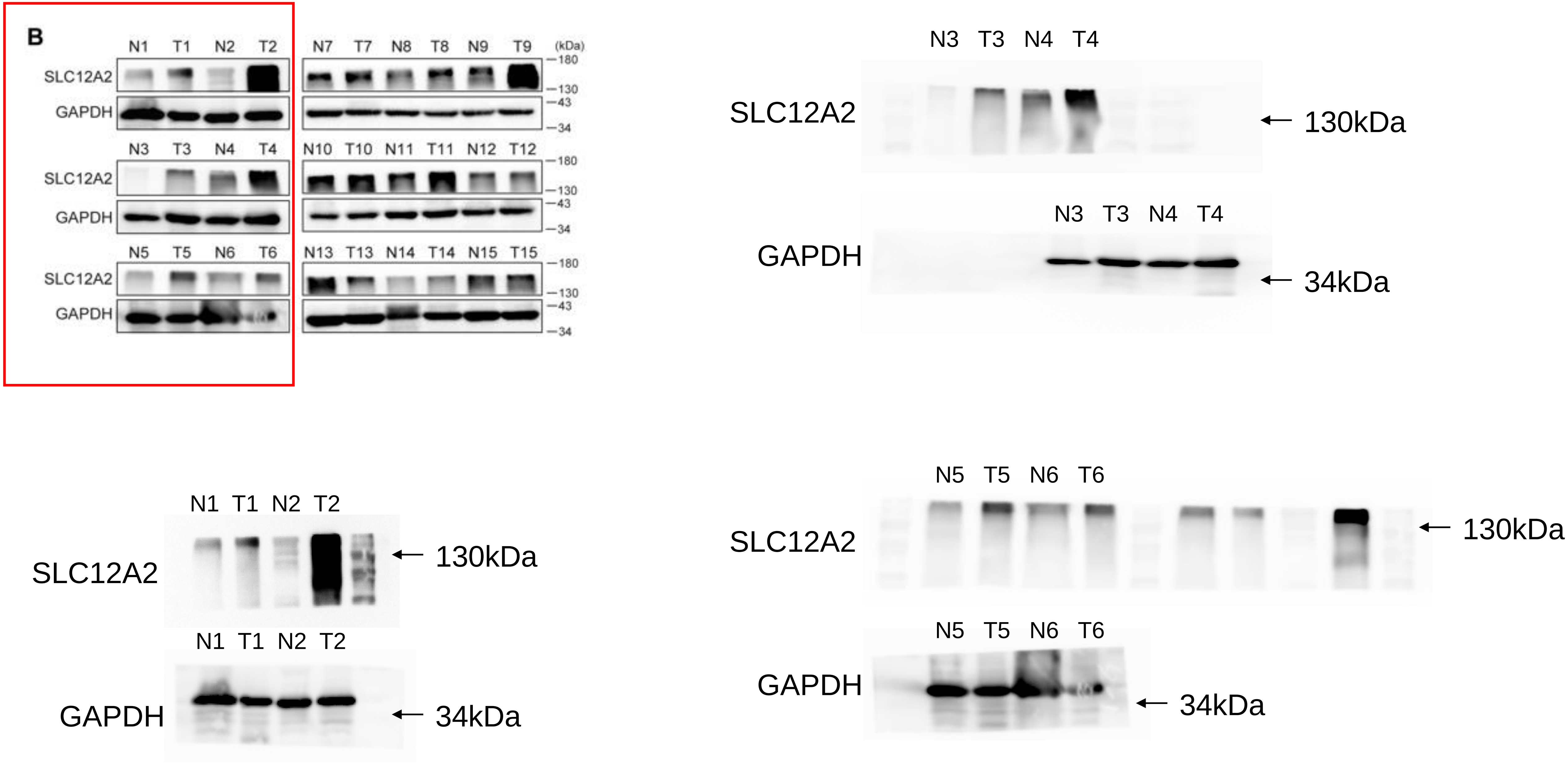
B
N1 T1 N2 T2
N7 T7 N8 T8 N9 T9
(kDa)
SLC12A2
GAPDH
N3 T3 N4 T4
N10 T10 N11 T11 N12 T12
N5 T5 N6 T6
N13 T13 N14 T14 N15 T15
180
130
43
34
N3 T3 N4 T4
SLC12A2
130kDa
GAPDH
34kDa
N1 T1 N2 T2
SLC12A2
130kDa
GAPDH
34kDa
N5 T5 N6 T6
SLC12A2
130kDa
GAPDH
34kDa

Figure 9B

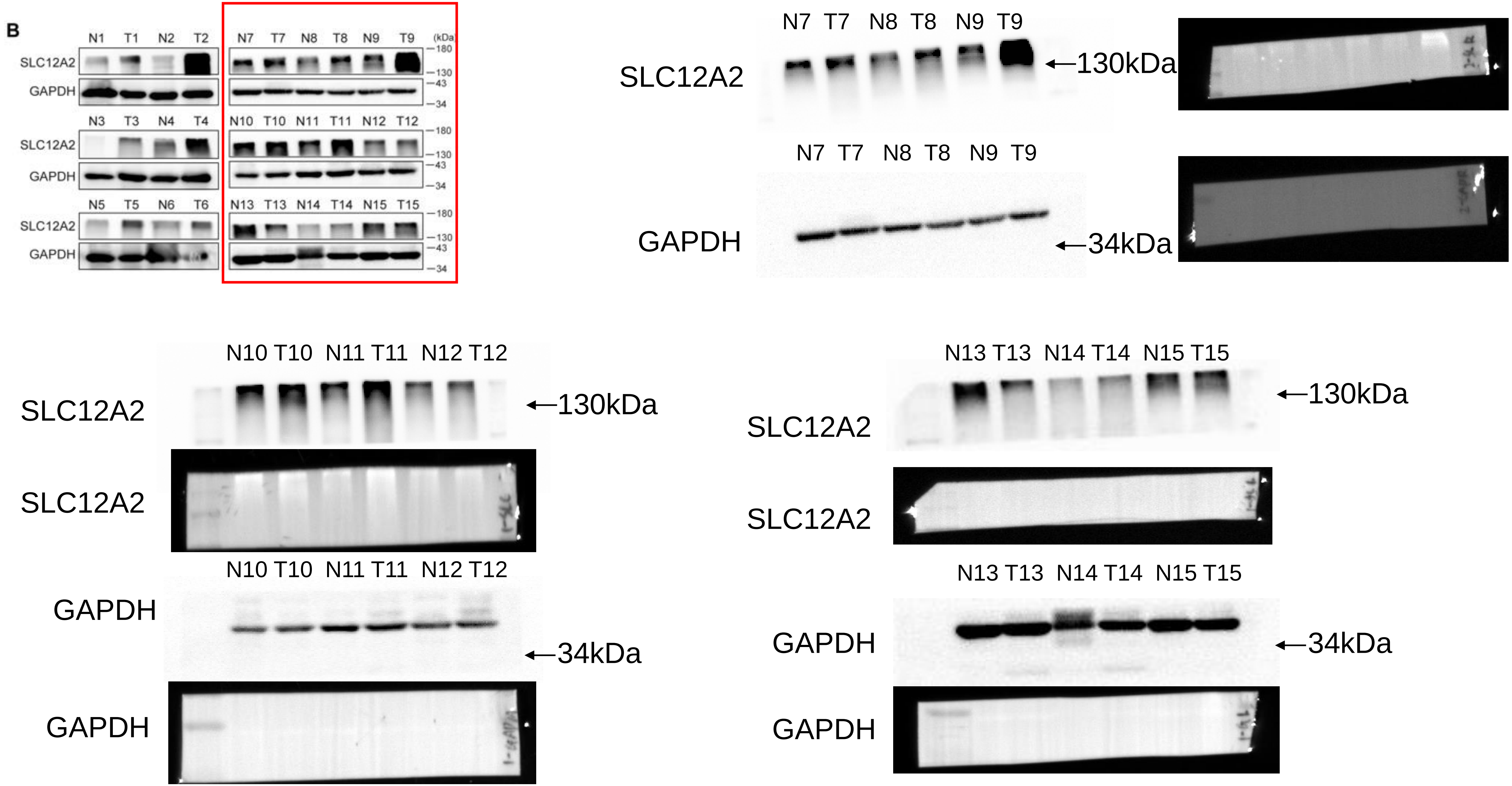
B
N1 T1 N2 T2
N7 T7 N8 T8 N9 T9
(kDa)
SLC12A2
GAPDH
N3 T3 N4 T4
N10 T10 N11 T11 N12 T12
N5 T5 N6 T6
N13 T13 N14 T14 N15 T15
180
130
43
34
N7 T7 N8 T8 N9 T9
SLC12A2
←130kDa
GAPDH
←34kDa
N10 T10 N11 T11 N12 T12
SLC12A2
←130kDa
GAPDH
←34kDa
N13 T13 N14 T14 N15 T15
SLC12A2
←130kDa
GAPDH
←34kDa

Figure 9D

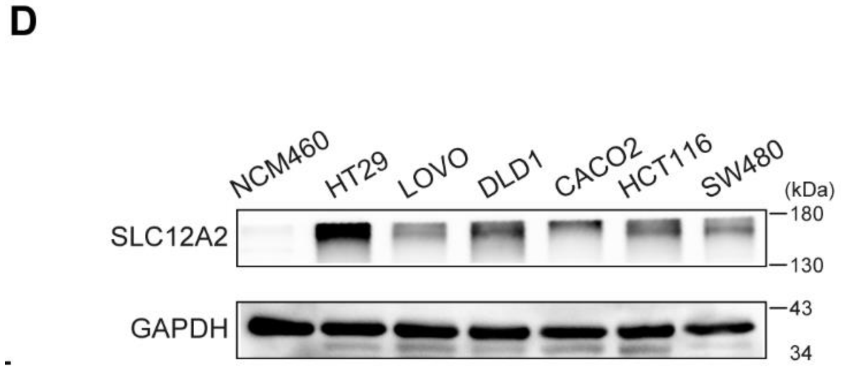
D
NCM460
HT29
LOVO
DLD1
CACO2
HCT116
SW480
(kDa)
SLC12A2
180
130
GAPDH
43
34

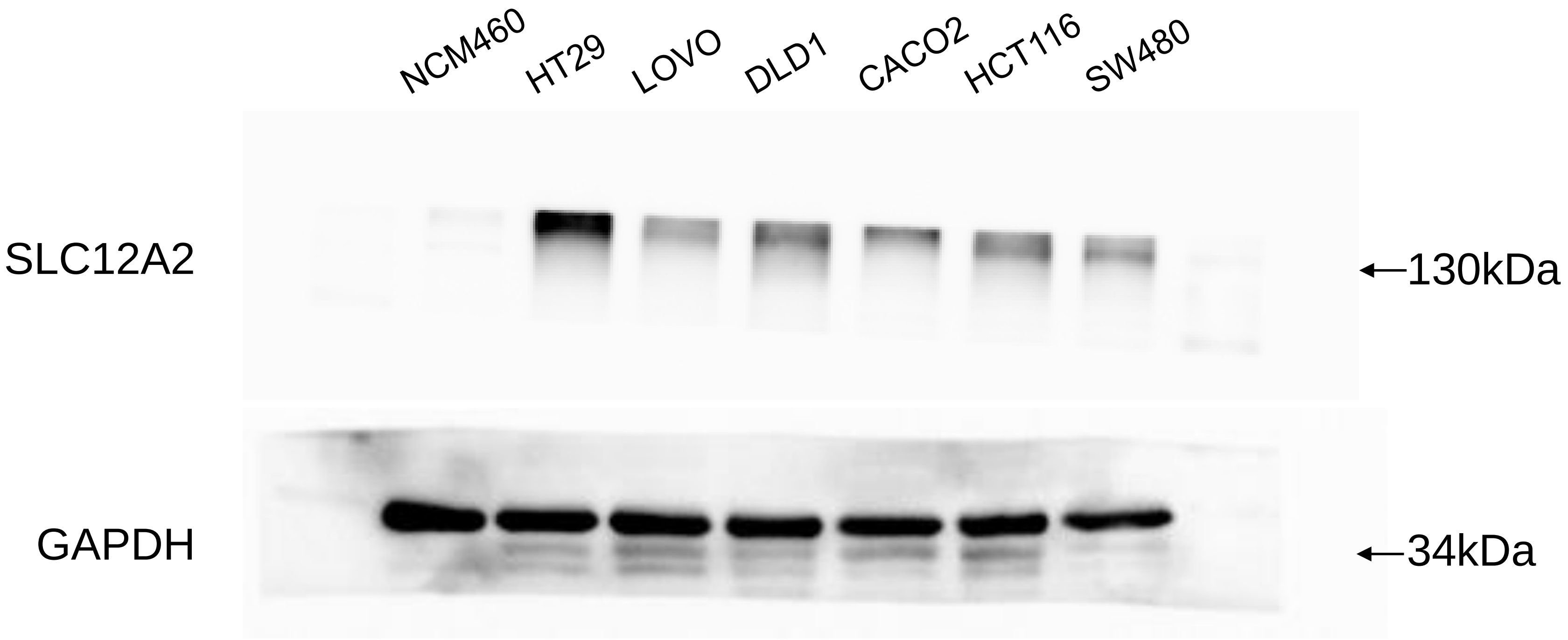
NCM460
HT29
LOVO
DLD1
CACO2
HCT116
SW480
SLC12A2
←130kDa
GAPDH
←34kDa

Figure 10E

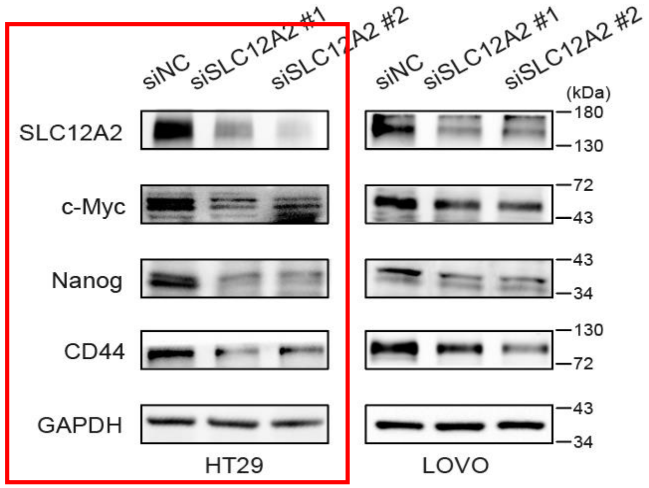
siNC
siSLC12A2 #1
siSLC12A2 #2
siNC
siSLC12A2 #1
siSLC12A2 #2
(kDa)
SLC12A2
c-Myc
Nanog
CD44
GAPDH
180
130
72
43
43
34
130
72
43
34
HT29
LOVO

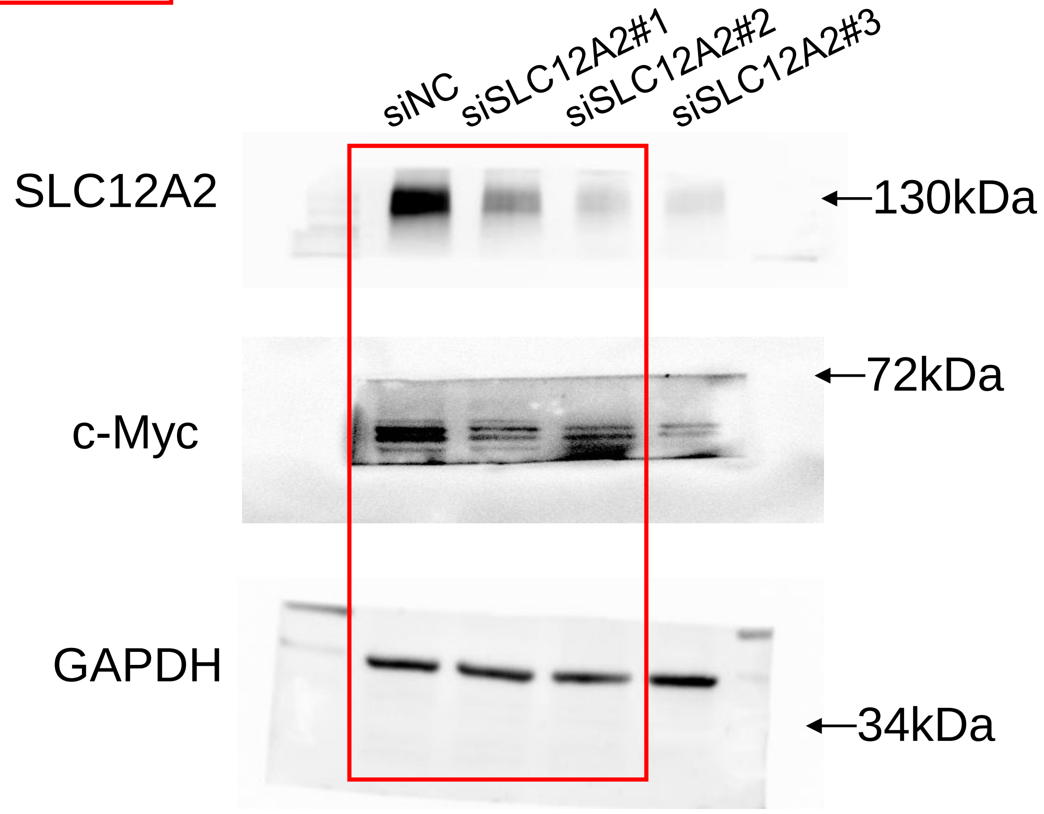
siNC
siSLC12A2#1
siSLC12A2#2
siSLC12A2#3
SLC12A2
c-Myc
GAPDH
130kDa
72kDa
34kDa

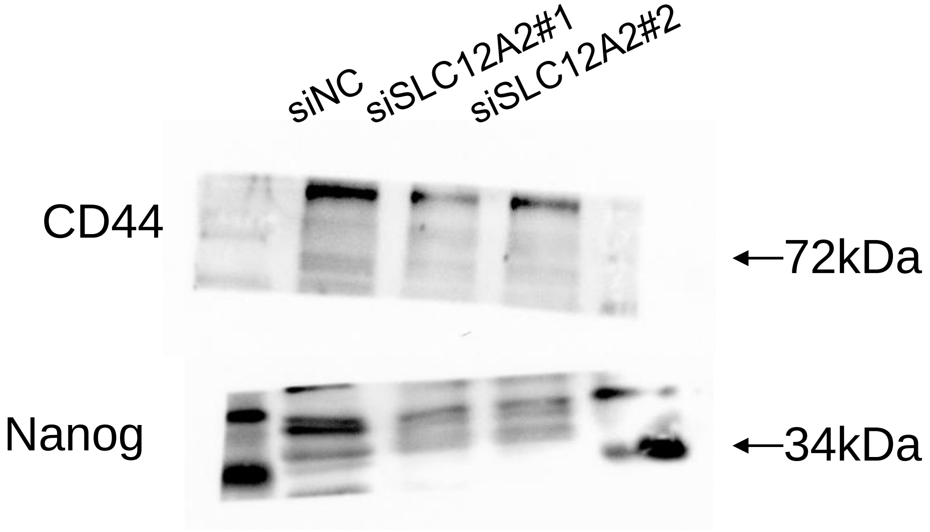
siNC
siSLC12A2#1
siSLC12A2#2
CD44
Nanog
72kDa
34kDa

Figure 10E

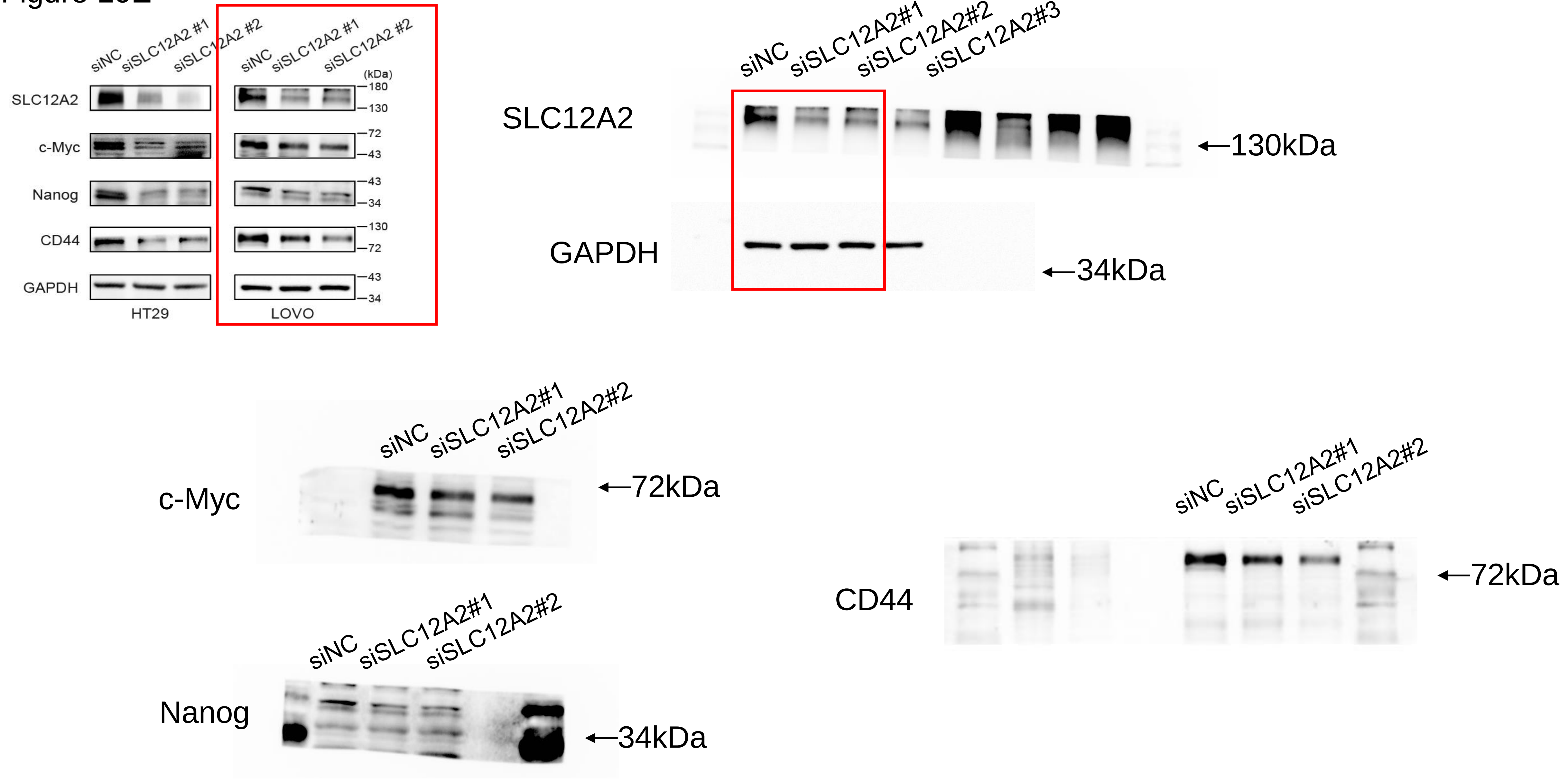
siNC
siSLC12A2 #1
siSLC12A2 #2
siNC
siSLC12A2 #1
siSLC12A2 #2
(kDa)
SLC12A2
180
130
c-Myc
72
43
Nanog
43
34
CD44
130
72
GAPDH
43
34
HT29
LOVO
siNC
siSLC12A2#1
siSLC12A2#2
siSLC12A2#3
SLC12A2
←130kDa
GAPDH
←34kDa
siNC
siSLC12A2#1
siSLC12A2#2
c-Myc
←72kDa
siNC
siSLC12A2#1
siSLC12A2#2
Nanog
←34kDa
siNC
siSLC12A2#1
siSLC12A2#2
CD44
←72kDa

Figure 10F

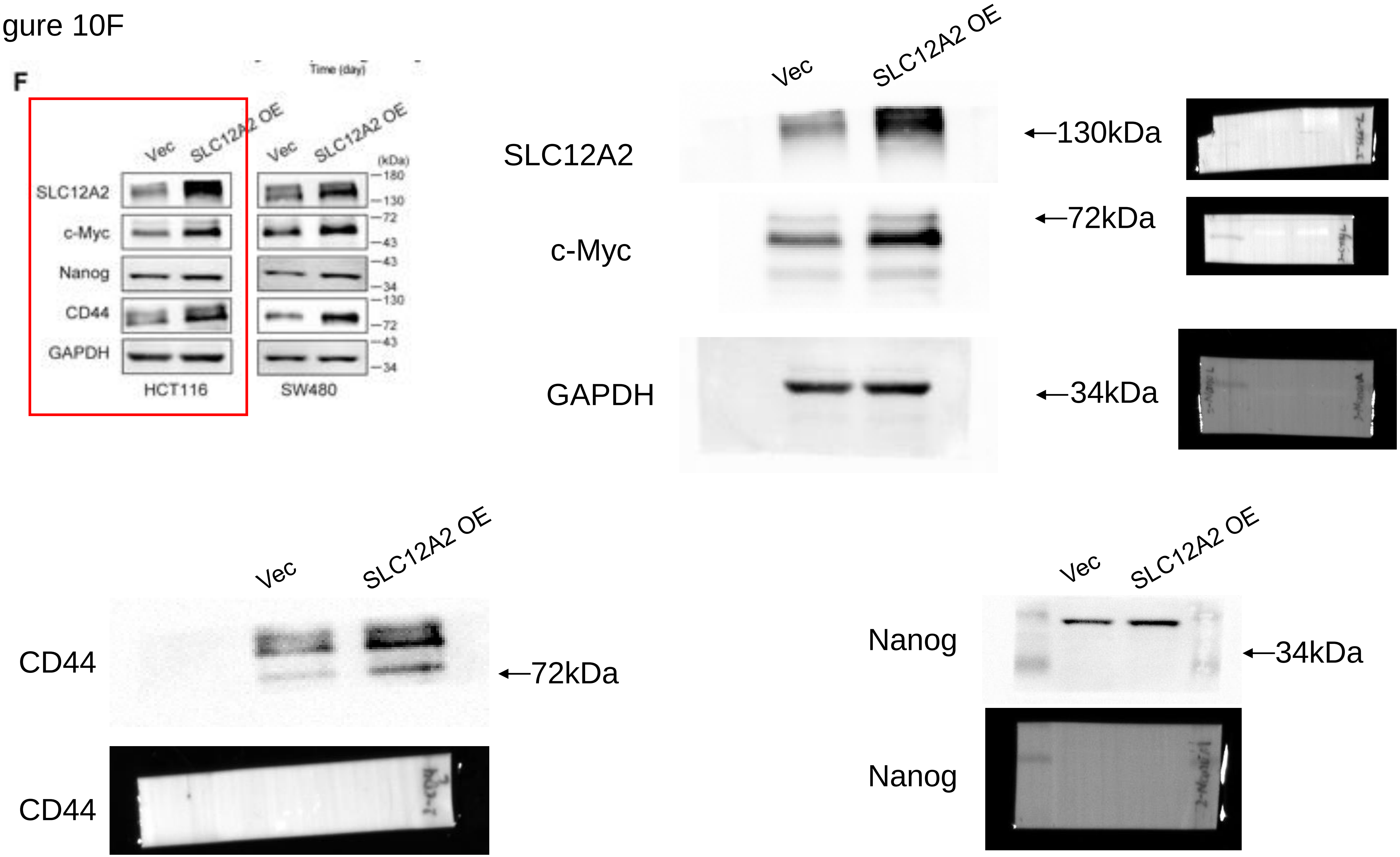

F
Time (day)
Vec
SLC12A2 OE
(kDa)
SLC12A2
c-Myc
Nanog
CD44
GAPDH
180
130
72
43
34
HCT116
SW480
←130kDa
←72kDa
←34kDa

Figure 10F

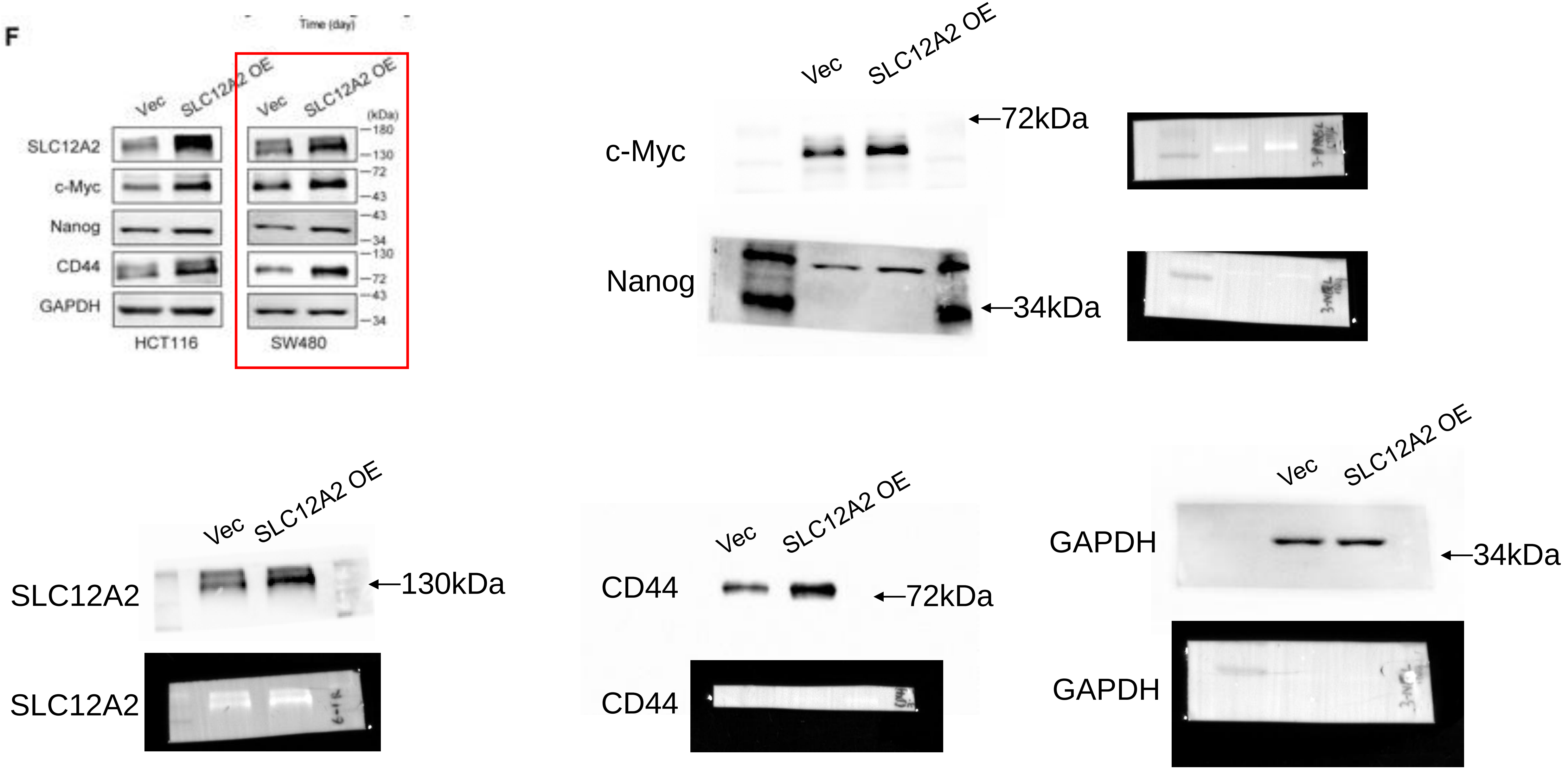
F
Time (day)
Vec
SLC12A2 OE
Vec
SLC12A2 OE
(kDa)
SLC12A2
c-Myc
Nanog
CD44
GAPDH
180
130
72
43
43
34
130
72
43
34
HCT116
SW480
Vec
SLC12A2 OE
c-Myc
←72kDa
Nanog
←34kDa
SLC12A2
←130kDa
SLC12A2
CD44
←72kDa
CD44
GAPDH
←34kDa
GAPDH
